# Poroelastic osmoregulation of living cell volume

**DOI:** 10.1016/j.isci.2021.103482

**Published:** 2021-11-22

**Authors:** Mohammad Hadi Esteki, Andrea Malandrino, Ali Akbar Alemrajabi, Graham K. Sheridan, Guillaume Charras, Emad Moeendarbary

**Affiliations:** 1 Department of Mechanical Engineering, Isfahan University of Technology, Isfahan, Iran; 2 Department of Mechanical Engineering, University College London, London, UK; 3 Biomaterials, Biomechanics and Tissue Engineering Group, Department of Materials Science and Engineering, Universitat Politècnica de Catalunya, Barcelona, Spain; 4 Department of Biological Engineering, Massachusetts Institute of Technology, Cambridge, MA, USA; 5 School of Life Sciences, Queens Medical Centre, University of Nottingham, Nottingham, UK; 6 London Centre for Nanotechnology, University College London, London, UK; 7 Department of Cell and Developmental Biology, University College London, London, UK; 8 Institute for the Physics of Living Systems, University College London, London, UK

**Keywords:** Cellular physiology, Cell biology, Biomechanics

## Abstract

Cells maintain their volume through fine intracellular osmolarity regulation. Osmotic challenges drive fluid into or out of cells causing swelling or shrinkage, respectively. The dynamics of cell volume changes depending on the rheology of the cellular constituents and on how fast the fluid permeates through the membrane and cytoplasm. We investigated whether and how poroelasticity can describe volume dynamics in response to osmotic shocks. We exposed cells to osmotic perturbations and used defocusing epifluorescence microscopy on membrane-attached fluorescent nanospheres to track volume dynamics with high spatiotemporal resolution. We found that a poroelastic model that considers both geometrical and pressurization rates captures fluid-cytoskeleton interactions, which are rate-limiting factors in controlling volume changes at short timescales. Linking cellular responses to osmotic shocks and cell mechanics through poroelasticity can predict the cell state in health, disease, or in response to novel therapeutics.

## Highlights

• Cell height changes can be finely captured by defocusing microscopy
• Water permeation and cellular deformability regulate dynamics of cell volume changes
• Poroelasticity describes the dynamics of cell volume changes
• The response of cell to hypo or hyperosmotic shocks are modeled by poroelasticity

## Graphical abstract

**Figure:**
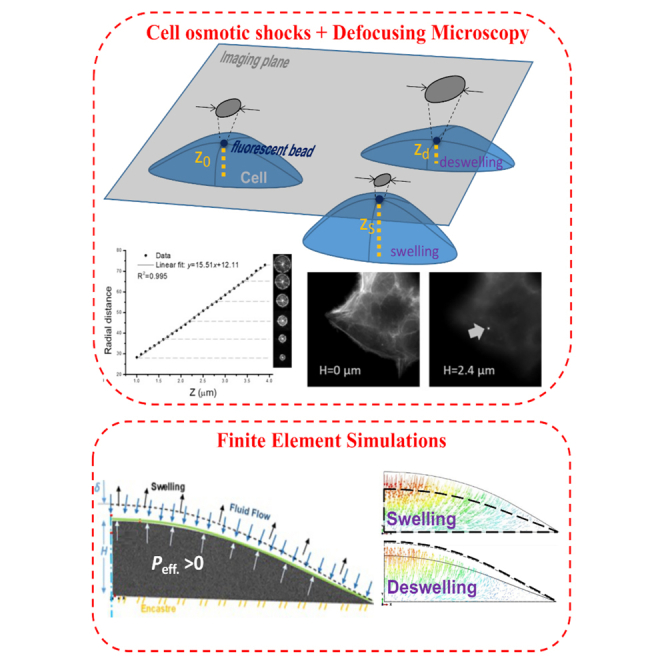

## Introduction

Cell volume regulation involves key homeostatic mechanisms that act in concert to maintain a well-defined intracellular ionic environment when external osmotic challenges arise (Chamberlin and Strange, 1989; Strange, 2004). For that, passive water and ion flow through membrane channels, together with active ion exchange via ATP-driven co-transporters, help to maintain cell volume in health and disease states (McManus et al., 1995; Cadart et al., 2019). Several mathematical models have been proposed to describe such volume adaptations (Hernández and Cristina, 1998; Lim et al., 2006; Solenov et al., 2011; Sachs and Sivaselvan, 2015; Cadart et al., 2019). The true complexity of volumetric responses displayed by cells exposed to external osmotic perturbations is not explained by modeling cells as simple water-filled receptacles composed of a lipid-rich plasma membrane (Fels et al., 2009). Instead, the mechanics of the cytoskeleton—a key determinant of cellular behavior, phenotype, and shape (Cosgrove, 1993; Ofek et al., 2009; Jiang and Sun, 2013)—is intimately linked with the volume of mammalian cells (Fernández and Pullarkat, 2010). Additionally, cell shape and volume are regulated by geometrical constraints, adhesive and mechanical interactions with extracellular environment (Fernández and Pullarkat, 2010; Ambriz et al., 2018), the rheological properties of cytoskeleton (Fernández and Pullarkat, 2010), the spatial distribution of water flux across the cell membrane, and hydraulic pressure acting at the cell surface (Moeendarbary et al., 2013; Han et al., 2020; Tao et al., 2017). In the context of tumor biology, for example, it has been shown that cancer cells positioned on the outer layers of tumor spheroids display larger volumes and are softer, conferring a more invasive phenotype compared to cells located within the tumor core (Han et al., 2020). Analogous to a fluid-filled sponge, a solid skeleton of the cell, consisting of cytoskeletal proteins, organelles, and macromolecules, is permeated by intracellular fluid. The dynamics of these fluid movements within the gelatinous cytoplasm can control the rate of adaptive cell volume changes under both hypoosmotic and hyperosmotic conditions (Strange, 2004; Moeendarbary et al., 2013).

Under the poroelastic framework, cells are treated as fluid-filled spongy materials, providing a more representative depiction of their inherent hydrogel-like composition (Fels et al., 2009; Moeendarbary et al., 2013; Gautier et al., 2015; Moeendarbary and Charras, 2015). Hydrogels are cross-linked polymer networks permeated by water molecules and their response to mechanical forces depends on the hydrodynamic interaction between the solid phase (e.g. in cells, the cytoskeleton) and the fluid phase (the cytosol). Indeed, the velocity of fluid redistribution through the pores of the solid meshwork and the deformability of the solid meshwork control the rate at which hydrogels can expand or shrink (Nam et al., 2004; Mitchison et al., 2008; Persson and Halle, 2008; Moeendarbary et al., 2013; Esteki et al., 2020). In addition to the hydrogel-like passive mechanical behavior, cells actively remodel their cytoskeleton at minute timescales, potentially influencing the dynamics of shape change (Li et al., 2018; Garenne et al., 2020; Rizzelli et al., 2020). On the one hand, fluid flows into a cell due to hypoosmotic conditions lead to cell swelling and increase the intracellular hydraulic pressure (Sardini et al., 2003; Wilson and Mongin, 2018). Consequently, the cell swelling may lead to changes in gene transcription via Ca^2+^ regulated mechanotransduction signaling pathways and relocalization of transcription factors (Okada et al., 2001; Nilius et al., 2003) as the cytoskeleton is connected to protein complexes that regulate the opening and closing of mechanosensitive ion channels (e.g. TRPV4). Swelling can also cause membrane blebbing and increase the metastatic potential of cancer cells (Khan et al., 2019). On the other hand, hyperosmotic stress and cellular shrinkage may decrease intracellular hydraulic pressure and lead to pathologies induced by either cytoplasmic stiffening (Prystopiuk et al., 2018; Han et al., 2020), DNA damage and apoptosis (Burg et al., 2007), oxidative stress and inflammation (Vaziri and Rodríguez-Iturbe, 2006), or cell-cycle arrest, and cellular senescence (Myers et al., 2007). Indeed, as cells age, the density and cross-linking of the cytoskeletal network (i.e. F-actin filaments) increases leading to a decrease in cytoskeletal pore size, slower fluid diffusion within old cells (Phillip et al., 2015), and cellular stiffening (Wilson and Mongin, 2018). As such, a poroelastic theory that mechanistically captures cell volume changes in response to osmotic changes can be used to make predictions regarding the biological significance of cell volume changes in different cell types (Mitchison et al., 2008; Moeendarbary et al., 2013; Sachs and Sivaselvan, 2015).

Pioneered by Terzaghi for the 1D settling of water-saturated soils (Terzaghi, 1925), and later generalized and extended in 3D by Biot (Biot, 1941, 1956), poroelasticity has been applied to study rheological behavior of hydrogels and more recently fluid flows and pressurization in cells (Hu et al., 2010; Chan et al., 2012; Moeendarbary et al., 2013; Esteki et al., 2020). Passive intracellular pressurization often arises from solute concentration differences between the extracellular and intracellular spaces, which are separated by a semipermeable cell membrane. In the absence of active ion transporters, the cell membrane allows solutions to equilibrate, that is, water permeates toward the solution of higher ionic concentration causing an extra pressure on the membrane defined as the osmotic (or turgor) pressure (Π) (Van’t Hoff, 1966; Liu et al., 2019a, 2019b). Application of hydrostatic pressure (p) difference across the cell membrane can also drive water movement. Therefore, the net pressure inside the cell (*P*_in_ = p_i_- Π_i_) and the net pressure outside the cell (*P*_out_ = p_o_-Π_o_) define the total effective pressure (*P*_eff._ *= P*_out_ - *P*_in_) across the cell membrane which drives the flow of water in or out of the cell. A positive effective pressure difference (*P*_eff._ >0) causes fluid to flow into the cell and swelling. Conversely, a negative effective pressure difference (*P*_eff._ <0) leads to shrinkage. The swelling or shrinkage involves the water flowing into or out of the cell by crossing the lipid cell membrane. While swelling or shrinkage depends on the water permeation that primarily relies on the membrane permeability as well as the hydraulic resistance of the cytoplasm, the rheological properties of the cytoplasm critically modulate the rate of the volume changes (Moeendarbary and Charras, 2015; Liu et al., 2019a, 2019b; Malandrino and Moeendarbary, 2019). Poroelasticity, as a coarse-grained bottom–up framework (Bausch and Kroy, 2006), can account for these factors and thus provides a mechanistic framework for understanding the swelling/shrinkage kinetics.

In recent swelling experiments, the mechanical behavior of several hydrogels and tissues has been characterized (Chandran and Barocas, 2004; Yoon et al., 2010; Bouklas and Huang, 2012; Gupta and Shivakumar, 2012; Bouklas et al., 2015; Wong et al., 2015; Castro et al., 2018; Tanska et al., 2020) through determining the poroelastic parameters by analytical solutions and investigating their effects on the transient and steady-state responses (Buckley et al., 2009; Yoon et al., 2010; Bouklas and Huang, 2012; Gupta and Shivakumar, 2012; Li et al., 2013; Bouklas et al., 2015; Caccavo et al., 2018; Yu et al., 2018). In these studies, it is assumed that initial pressurization ramps instantaneously (i.e. rise time t_r_ = 0), while an instantaneous pressure rise assumption is mostly unrealistic under experimental circumstances and may lead to incorrect estimation of the swelling/deswelling poroelastic parameters during a transient osmotic stimulus (Javanmardi et al., 2021). Moreover, active processes at the cell membrane (i.e. cellular regulatory volume processes via ion channels) and osmotic and hydrostatic challenges that cells experience in different physiological and disease states may create gradual effective pressure differences, that is, with a finite rise time. It has been concluded that neglecting the effects of finite rise times (t_r_) during the analysis of the indentation of poroelastic materials may lead to unrealistic estimations of poroelastic parameters (Oyen, 2008; Galli and Oyen, 2009; Hu and Suo, 2012; Mollaeian et al., 2017; Mollaeian et al., 2018a, 2018b; Liu et al., 2019a, 2019b; Esteki et al., 2020). However, the influence of finite pressure rise-time associated with osmotic and hydrostatic challenges on the realistic evaluation of swelling/deswelling dynamics has not been considered yet.

Here, we employed a combination of cell culture experiments, high-resolution nanoparticle tracking, analytical solutions, and finite element (FE) simulations to investigate the poroelastic swelling/deswelling behavior of adherent cells in response to transient pressure differences caused by osmotic challenges. We measured the dynamics of HeLa cell swelling and deswelling by defocusing microscopy and ran a series of analytical calculations and FE simulations to identify critical poroelastic parameters, including geometrical and rise times effects. In our modeling framework, considering hydraulic (${\text{p}}_{\text{o}}-{\text{p}}_{\text{i}}$) and osmotic (${\text{Π}}_{\text{o}}-{\text{Π}}_{\text{i}}$) pressures, we defined the effective pressure difference as ${\text{P}}_{\text{eff}.}$ = $\left({\text{p}}_{\text{o}}-{\text{p}}_{\text{i}}\right)$–$\left({\text{Π}}_{\text{o}}-{\text{Π}}_{\text{i}}\right)$ , which is a net driving pressure for cellular volumetric deformations. This phenomenological approach has the advantage of being directly implementable in the FE simulations. Considering a realistic 3D cell geometry, we investigated the poroelastic responses of cells under finite pressure rise-time. Our results indicate that dynamics of cell volume changes at short timescales can accurately be described by the poroelasticity framework and our estimated cellular poroelastic parameters are consistent with previously reported values.

## Results

### Experimental results

**Figure 1:**
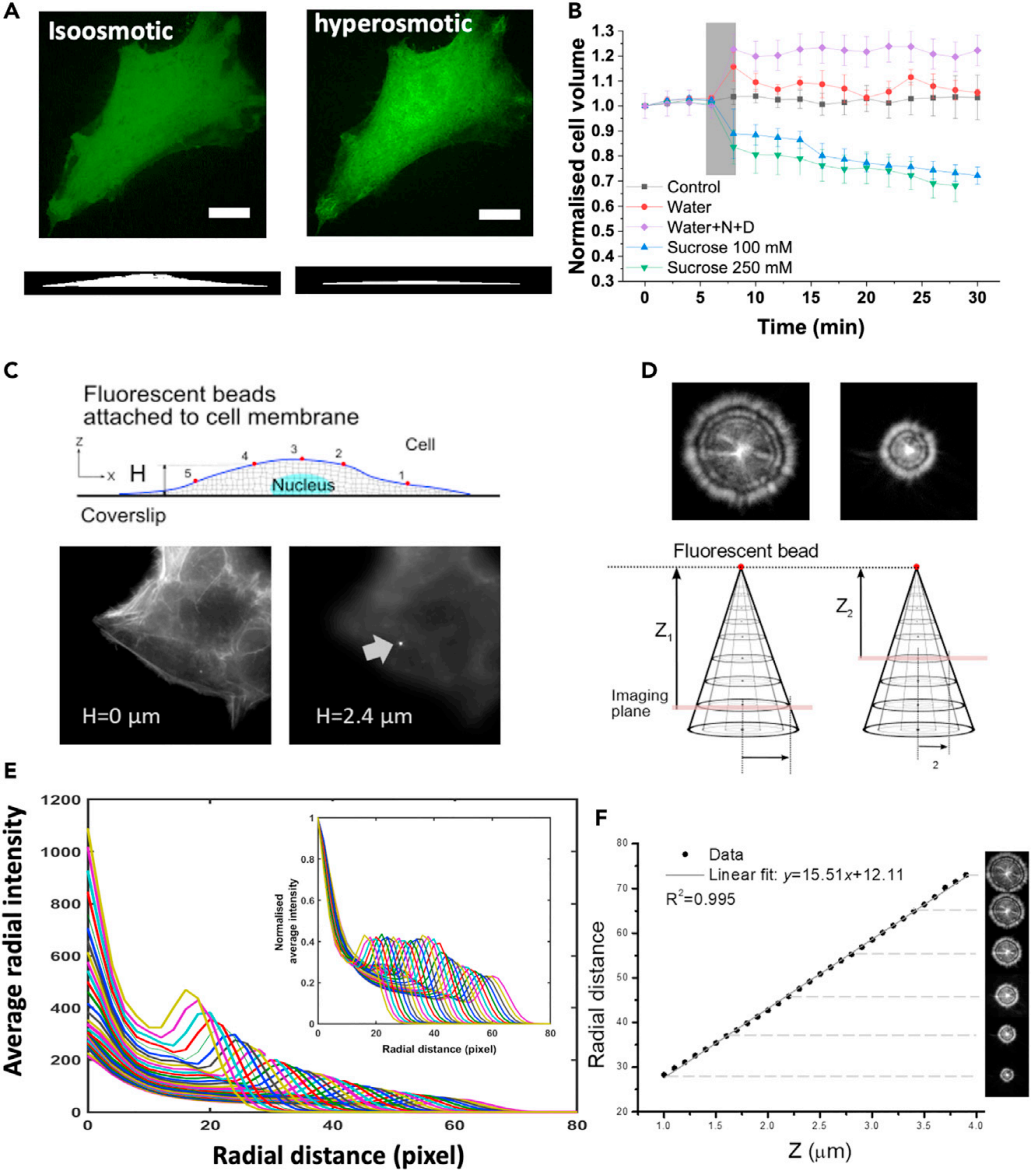
Experimental measurement of cell volume and of the temporal evolution of cell height (A) The volume of HeLa cells was measured by acquiring xyzt confocal stacks. Top images show the cytoplasmic GFP cells under isosmotic and hyperosmotic conditions and the bottom images are orthogonal views of the 3D reconstruction of the cell volume. Scale bars = 10μm. (B) Cell volume change over time in response to changes in extracellular osmolarity. Error bars indicate the standard deviation from n = 3–5 independent experiments. The shadow area indicates the time of addition of osmolytes. The water + N + D and sucrose 100mM curves are extracted from our previous work (Moeendarbary et al., 2013). Error bars indicate the standard deviation. (C) Schematic of fluorescent nanobeads covalently linked to the cell membrane. The height of a bead attached to the cell is estimated by first finding the z position of the bottom of the dish (shown in the left focused image) where stress fibers are in focus, and then the position for which the bead attached to the cell membrane is in focus (shown in the right focused image). (D) Keeping the focal plane fixed, the change in vertical position of each bead after swelling/shrinkage was determined from the radius of the outer ring formed in the defocused image. (E) Moving the imaging plane (red line in panel d) in 100 nm increments away from the bead focal plane creates a series of curves showing the averaged radial fluorescence intensity as a function of distance from the center of the nanobeads. Inset shows the averaged radial intensity curves normalized to the maximum intensity in their center. (F) Corresponding calibration curve demonstrating the linear relationship between the radial distance of outer ring and the height (z-position). The radial position of the ring (peak intensity) is linearly related to the distance between the imaging plane and the nanobead focal plane and therefore the z-position of the nanobeads can be tracked with very high accuracy (∼10 nm).

The temporal evolution of the volume of HeLa cells in response to osmotic shocks was measured by acquiring fast xyzt spinning disk confocal stacks. Figure 1A shows an example of orthogonal profiles of a HeLa cell before and after application of a hyperosmotic shock. The decrease in cell volume resulted in an increase in fluorescence intensity indicating the increase in GFP concentration due to water expelling. The 3D reconstruction of cell profile from z-stacks before and after application of the shock displayed a substantial cell height decrease (Figure 1A, bottom projections) suggesting that probing the cell height is a good indicator of the volume changes. We then measured cell volume changes in response to changes in osmolarity for up to 30 min to ascertain that volume changes persisted long enough for our AFM indentation measurements (Figure 1B). We verified that photobleaching due to the acquisition of confocal stacks over an extended period did not artefactually affect our measurement of cell volume (control, Figure 1B). To ensure a stable volume increase in hypoosmotic conditions over the timescale necessary for AFM experiments (∼30 min), during measurements, we added regulatory volume decrease inhibitors to the medium (Lang et al., 1998). We measured an unstable cell volume increases up to 10% upon addition of water for hypoosmotic treatment while a stable cell volume increase of 22 ± 2% was achieved upon addition of water + N + D. Conversely, upon addition of 100 and 250 mM sucrose, cell volume decreased to 12 ± 3% and 21 ± 6% of the original volume, respectively.

We maximized the rate of application of the osmotic shocks by exchanging the media of different osmolality immediately (over less than a few seconds). Our results indicate that most volume changes occur within the first minute following the exchange of the media (Figure 1B). Furthermore, employing spinning disk confocal z-stacks and image processing algorithms, we estimated errors of at least 20% associated with capturing cell height (i.e. 400 nm is the best resolution we could achieve in z direction using confocal microscopy). Also, the maximum rate at which we could acquire the stacks covering a whole cell was ∼1 min. Taken together, despite spinning disk confocal imaging providing a good estimation of volume changes over long timescales (tens of minutes), it is not possible to capture the full transitory dynamics of cell height changes (occurring at millisecond to second timescales) with this technique.

**Figure 2:**
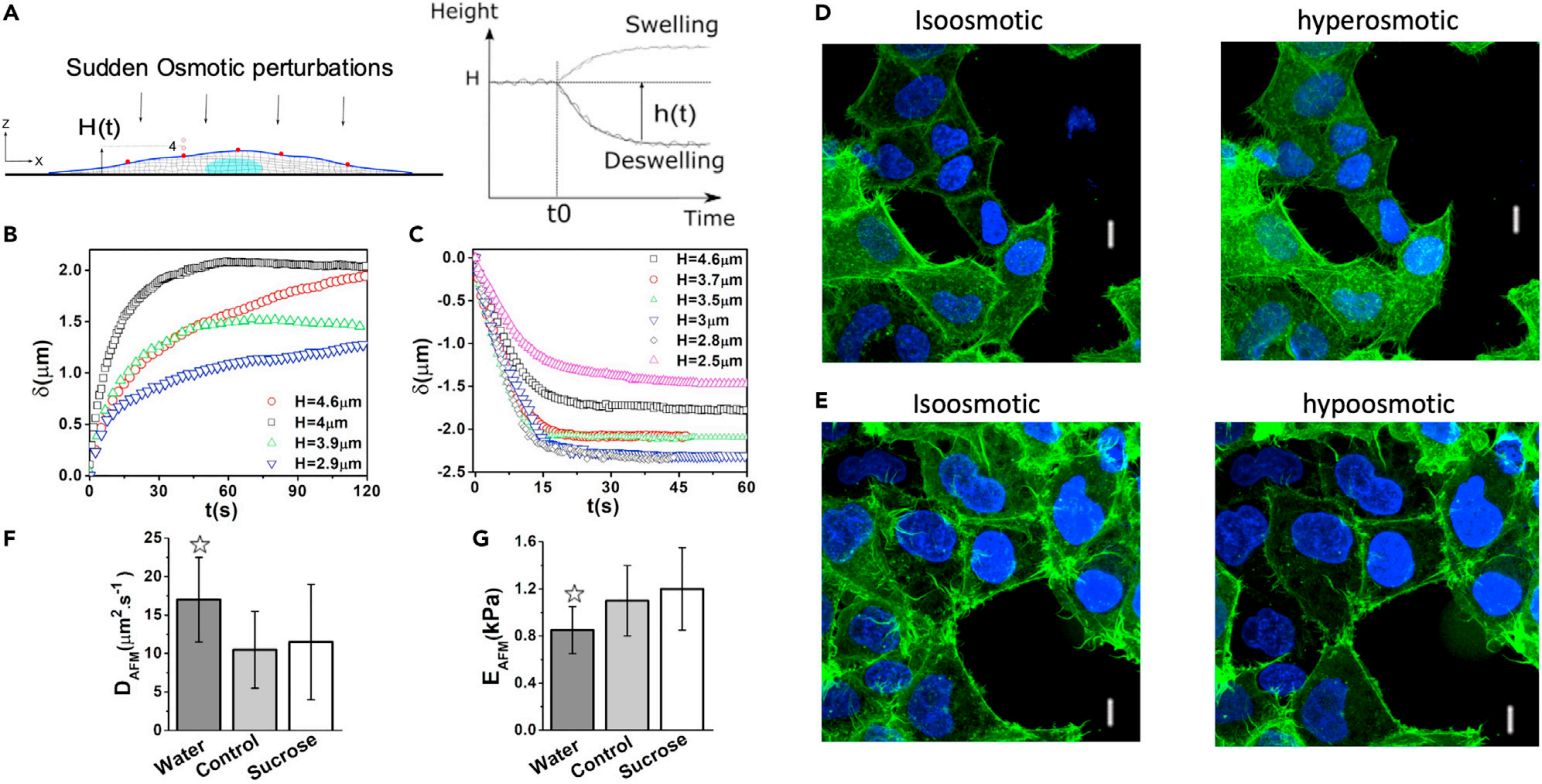
Swelling/deswelling experiments and effects of hypo or hyperosmotic shock on F-actin organization (A) By changing the osmolarity of the extracellular medium, osmotic pressure gradients were induced across the cell membrane. The pressure gradient direction (hypo or hyperosmotic shock) drives water in or out of the cell causing swelling or deswelling (a deswelling case is depicted in this schematic) and changes in bead height. (B and C) Temporal changes in the height of HeLa cells under hypoosmotic (B) and hyperosmotic (C) shocks. (D and E) Images of F-actin structures before and after 30 min application of osmotic perturbation. Images are maximum projections of confocal stacks of cells expressing Life-act ruby (green). Nuclei were stained with Hoechst 34,332 (blue). Scale bars = 10μm. (F and G) The diffusion constant and elastic modulus of cells obtained by AFM indentation tests on cells after application of osmotic changes. Error bars indicate the standard deviation in all graphs. To test pairwise differences in population experiments, Student's t-test was performed and values of p < 0.01 were considered significant and are indicated by stars.

To address the limiting factors (i.e. lack of spatial and temporal resolutions) associated with typical confocal 3D imaging and capture the short timescale dynamics (shaded area in Figure 1B), we employed epifluorescence defocusing microscopy (see STAR Methods). We exposed HeLa cells to hyperosmotic or hypoosmotic medium at t_r_∼0 while tracking the vertical displacement of fluorescent nanobeads as a function of time and analyzing the defocused 2D images taken at 100 ms intervals (Figures 1D and 2A). The changes in the thickness of the cells *δ* at the bead positions (located at specific heights) as a function of time are plotted in Figures 2B and 2C for swelling and deswelling conditions. In swelling experiments, most vertical displacements were larger for greater cell heights while this trend was weaker in deswelling cases. Furthermore, the timescale associated with deswelling was smaller and most curves reached a stable plateau after 20s compared to 50s for swelling curves that reach a less stable plateau. These observations suggest the inherent differences in the dynamics of cell swelling and deswelling that mainly originate from different mechanical responses of the cell membrane, cytoskeleton, and other cellular structures (nucleus, organelles, macromolecules) to stretch or compression (Isenberg et al., 2003; Chao et al., 2018). When normalizing the vertical displacements δ (Figures 2B and 2C) to their respective maximum final vertical displacement *δ*_∞_ (Figures S3A and S3B), we noticed distinct dynamics with varied responses of different positions on the cell surface and also at short and long timescales. Interestingly, the normalized displacement curves did not collapse on each other and therefore, we asked whether this observation is, in principle, valid when considering the cell to be an ideal poroelastic material.

We also investigated the effects of hypoosmotic or hyperosmotic shocks on the cytostructural organization of the HeLa cells and particularly F-actin which is the main determinant of the cell mechanical properties. The F-actin cytoskeleton did not reorganize in response to hyperosmotic treatment (Figure 2D) or hypoosmotic shock (Figure 2E), as the density and morphology of actin filaments were not noticeably altered. These observations, together with the contribution of intrinsic viscoelasticity of filaments (arising from cytoskeletal reorganization) to relaxation times at long timescales (over 60s), suggest that considering a purely elastic solid skeleton at short timescales (less than 60s) is a well-justified consideration in our poroelastic model.

To measure the elastic modulus required in the estimation of poroelastic parameters in osmotic experiments, we conducted AFM indentation tests (see STAR Methods). Additionally, these indentation tests provided an independent way of measuring poroelastic diffusion constant (Hu et al., 2011; Chan et al., 2012; Esteki et al., 2020) and showed how changes in cell volume influence the poroelastic parameters. In the case of swelling, addition of water increases cell volume and significantly increases (55%) the poroelastic diffusion constant and decreases (23%) cellular elastic modulus (Figures 2F and 2G).

### Poroelastic analysis of simple 2D geometries

**Figure 3:**
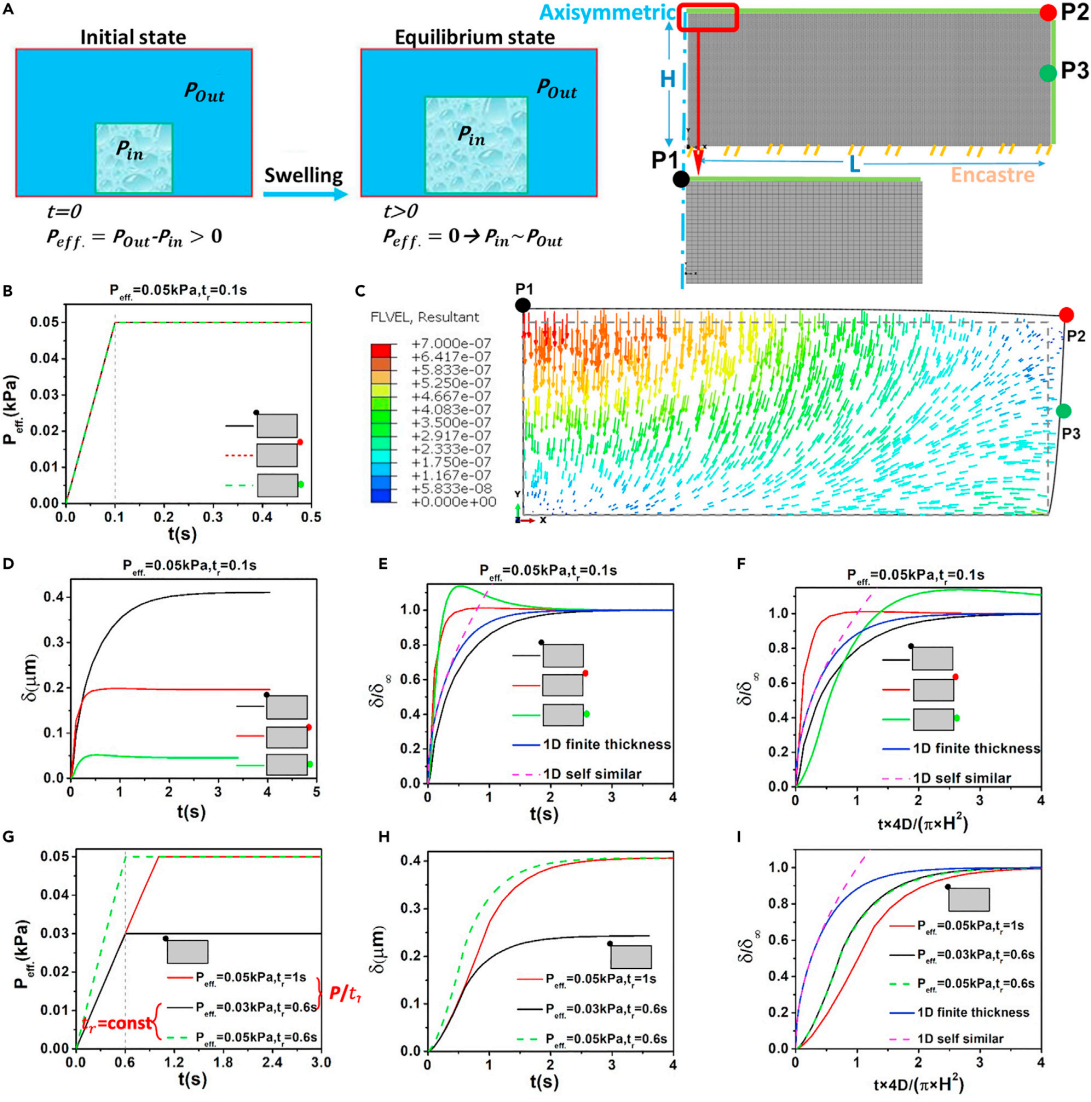
Effects of spatial location, rise time, effective pressure for constrained swelling of a simple geometry investigated by FE linear poroelastic simulations (A) Idealised swelling of a thin layer of a poroelastic material attached from one side to a substrate and initially at equilibrium $P_{eff.}$.∼ *P*_*out*_*- P*_*in*_*= 0*. Axisymmetric FE model of a thin disk with thickness H and length L was built to simulate effective pressure-driven swelling considering $P_{eff.}$ > 0 → *P*_*in*_ < *P*_*out*_. Vertical displacements of specific nodes located at different positions P1, P2, and P3 were studied. (B) Linear increase of effective pressure from 0 to $P_{eff.}$ = 0.05 kPa over a rise time *t*_r_ = 0.1s. (C) The swelling type deformation of the hydrogel due to application of pressure difference and inflowing of water. Dash and solid lines indicate non-deformed and deformed material boundaries respectively. The fluid velocity field is distributed non-uniformly at boundaries and within the domain and induces a non-uniform swelling behavior across the geometry. (D) Plot of vertical δ over time considering the ramp in pressure indicated in (B). (E) Normalization of (D) considering $\frac{\delta_{\;}}{\delta_{\infty \;}}$ for y axis and plots of 1D finite thickness and self-similar solutions. (F) Normalization of (D) considering $\tau =\frac{4Dt}{\pi H^{2}}$ for x axis and $\frac{\delta_{\;}}{\delta_{\infty \;}}$ for y axis. (G) Application of ramp with two different $P_{eff.}$ and two different $\;t_{r}$. (H) Considering conditions in (G), the plots of vertical displacement δ vs time were extracted for position P1. (I) Normalization of both x and y axis led to the overlaying of the curves when ${\left(\frac{P_{eff.}}{{\delta_{\infty \;}}_{\;}}\right)}_{1}={\left(\frac{P_{eff.}}{\delta_{\infty \;}}\right)}_{2}.\;$ The analytical solutions cannot fit any of the normalized curves.

We first considered a rectangular geometry and linear poroelastic FE model (Figure 3A and Table S1) to investigate swelling/deswelling in response to osmotic perturbations that were modeled by imposing an effective pressure *P*_eff._ boundary condition that linearly reaches its final value over the rise time *t*_*r*_ (Figure 3B). The rise time can be interpreted as the time of pressurization during deswelling, or depressurization during swelling. The consideration of rise time in our simulation is a new attempt to phenomenologically capture the effects of active membrane processes that make the cell membrane a time-dependent barrier to the transport of molecules and water in or out of the cell (Yamamoto et al., 2014; Yang and Hinner, 2015; Li et al., 2016; Emami et al., 2018; Yang et al., 2019). Furthermore, cells are not exposed to stepwise, instantaneous osmotic perturbations *in vivo* (Haskew-Layton et al., 2008; Pilizota and Shaevitz, 2012), and in our *in vitro* experiments, it takes at least a few seconds before cells are fully exposed to the media with different osmolarities despite our efforts to exchange the media immediately.

We tested the spatial heterogeneity of swelling/deswelling dynamics as the experimental vertical displacement curves differed largely for different positions (Figures 2B and 2C). We considered a set of poroelastic properties in the range of our AFM cellular measurements (*E* = 1kPa, *D* = 10μm^2^s^−1^) and effective pressure of 50 Pa which induces deformations at the scale of the experimental osmotic perturbations. Figure 3C shows swelling deformations and local fluid velocities predicted from the linear poroelastic FE model considering a short rise time of *t*_r_ = 0.1s (Figure 3B) and the constrained (no-slip condition for the bottom surface) boundary condition (see Figure S4A for constrained deswelling and Figure S6A for free swelling). Different local fluid velocities (Figures 3C and S4B) were observed at different positions of the rectangular geometry (P1, P2, and P3) leading to spatially different vertical displacements curves (*δ* vs *t*) for both swelling/deswelling conditions (Figures 3D and S4C). Also switching from no-slip to sliding boundary condition led to significantly different vertical displacement dynamics (Figure S6). To better compare constrained and free swelling, the velocity field and deformations for different boundary conditions are shown in Figure S8. Analysis of deformations and fluid profiles, suggests that a positive pressure gradient causes fluid entrance and swelling deformation (Figures S8A and S8C), while the negative pressure gradient induces expulsion of fluid and deswelling/shrinkage (Figures S8B and S8D).

Similar to the experimental observations (Figures S3A and S3B), normalization of vertical displacements with respect to their respective maximum final vertical displacement, $\delta_{\infty }$, did not lead to collapse of curves (Figures 3E and S4D) particularly at short time scales (t < 1 s). This demonstrates that different points at the cell surface can show unique poroelastic dynamics depending on their location relative to the boundaries. In Figure 3E, we also plotted the 1D finite thickness (Equation 25) and self-similar (Equation 26) analytical solutions considering stepwise ramp (considering the same poroelastic properties as in our FE simulations and H = 5.5 μm). The self-similar solution only captures the dynamics of deformations at very short time scales (t<<0.2s), while the finite thickness solution roughly follows the dynamics for the P1 position, which is the point that experiences the most symmetrical condition and the least boundary effects. Furthermore, normalization of time by $\tau =\frac{4Dt}{\pi H^{2}}$ based on Equation 26 and previous reports (Yoon et al., 2010; Bouklas and Huang, 2012)) did not improve overlaying of the curves (Figure 3F).

We next studied the sensitivity of the model predictions to *t*_*r*_ and *P*_eff_ considering three different scenarios: (i) *P*_eff._ = 0.03 kPa and *t*_*r*_ = 0.6s, (ii) *P*_eff._ = 0.05 kPa and *t*_*r*_ = 1s and (iii) *P*_eff._ = 0.05 kPa and *t*_*r*_ = 0.6s (Figure 3G). Figures 3H and S4G shows the *δ*-*t* curves (at position P1) for swelling and deswelling cases and suggest that for a fixed maximum pressure (*P*_eff._ = 0.05 kPa) but different rise times (*t*_*r*_ = 1s or 0.6s), the shape of *δ*-*t* curves is different particularly at short-time scales. This is mainly due to different fluid redistribution in the early phases of pressure relaxation. At longer time scales, the relaxation curves tend to overlay each other and plateau to a stable value of $\delta_{\infty }$ at time *t*_*∞*_. In swelling, a stable value of $\delta_{\infty }$ ∼0.4 μm was reached in *t*_*∞*_∼4 s while for deswelling, it took *t*_*∞*_∼3.5 s for $\delta_{\infty }$ ∼0.4 μm (Figures 3H and S4G). Interestingly, further normalization of time using $\frac{4Dt}{\pi H^{2}}$ illustrates that curves with equal values for both $\frac{P_{eff.}}{{\delta_{\infty }}_{\;}}$ and *t*_*r*_ fully overlay onto each other (Figures 3I and S4H), indicating the unique dependence of poroelastic dynamics to both time and length scales. Similar results were obtained by employing a porohyperelastic model (Figure S5), confirming the suitability of porohyperelasticity for application to more complex settings such as those we investigated in the next section. Moreover, we additionally examined the effects of non-linear increase in the P_eff._(t_r_) and confirmed that the height dynamics did not change (Figure S9) compared to the simple linear ramp function, which is assumed throughout the rest of the simulations.

Taken together, Figures 3E, 3F, and 3I (and Figures S4D, S4E, S4H, and S4I) highlight the noncompliance of analytical solutions with FE simulations, primarily due to different geometrical and boundary conditions. In fact, both analytical solutions (i.e. self-similar and finite thickness 1D models) assume simple 1D boundary with parameters that depend only on one direction while in our FE model flow and pressure distributions are multidirectional. Moreover, consideration of instantaneous application of pressure in analytical solutions versus finite rise time in FE simulations is another major reason for different solutions. The timescale of pressurization has been ignored in several recent studies (Rice and Cleary, 1976; Yoon et al., 2010; Bouklas and Huang, 2012; Li et al., 2013; Bouklas et al., 2015; Tanska et al., 2020) while here, it appears to be of fundamental relevance. Indeed, during the deformation of a poroelastic material, the key mechanism for fluid pressure changes relies on the ability of the interstitial fluid to redistribute through the pores of the solid phase. The distinctive feature of the poroelastic mechanical behavior is that the length scale l and the timescale t of pressurization or relaxation are correlated through $t_{p}∼l^{2}/D$, where *D* is the poroelastic diffusion coefficient (Hu et al., 2010; Yoon et al., 2010; Bouklas and Huang, 2012; Kalcioglu et al., 2012; Esteki et al., 2020). The presence of a characteristic length scale l is thus a unique feature of poroelastic mechanical responses. In our study, the length scale for both the pressurization and the subsequent fluid relaxation is the material thickness l∼H. Here, we propose a limiting case when this length scale and the poroelastic diffusion constant are such that $t_{r}$ and $t_{p}∼H^{2}/D$ are of the same orders, that is, when pressurization and volume changes occur on similar timescales. On the contrary, our model reconciles with previous analyses (Hu et al., 2010; Yoon et al., 2010; Bouklas and Huang, 2012; Kalcioglu et al., 2012; Esteki et al., 2020) in when $t_{r}$ is significantly shorter than $t_{p}$, $t_{r}\ll t_{p}∼H^{2}/D$.

To examine the effects of different permeabilities of membrane and cytoplasm, we simulated the membrane by considering an additional top thin layer (500nm thickness) with different diffusion coefficient compared to the base layer (Figure S10A). Indeed, the overall fluid redistribution can be captured by considering an apparent diffusion coefficient D that relies on both the diffusion coefficient of the membrane (D_2_) and the diffusion coefficient of the intracellular space (D_1_). No-slip condition between the two layers was assumed and other conditions were set similar to the single-layer FE model. We considered 2 sets of poroelastic properties (D_1_ = 10μm^2^s^−1^, D_2_ = 10, 1, 0.1μm^2^s^−1^ and D_1_ = 1μm^2^s^−1^, D_2_ = 1, 0.1, 0.01μm^2^s^−1^) and an effective pressure increase of 50 Pa. Figures S12C and S12E show vertical displacements curves predicted by the linear poroelastic FE model considering a short rise time of t_r_ = 0.1s at position P1. Varying membrane diffusion coefficient led to different vertical displacement curves (*δ* vs t). Interestingly, we found a correspondence between the use of different t_r_ in the one-layer FE model (t_r_ = 0.1, 0.6, 6s in Figures 3D and 3H) and different membrane diffusion coefficients (Figures S10C, and S10E). These results suggest that considering different diffusion coefficients for the cell membrane changes the shape of *δ*-t curves (Figures S10C and S10E) in a similar fashion as when changing rise time in the single-layer FE model. Therefore, considering a finite rise time in a single layer model may provide a simple way of mimicking the membrane effects.

### Toward more realistic analysis for cellular setting

**Figure 4:**
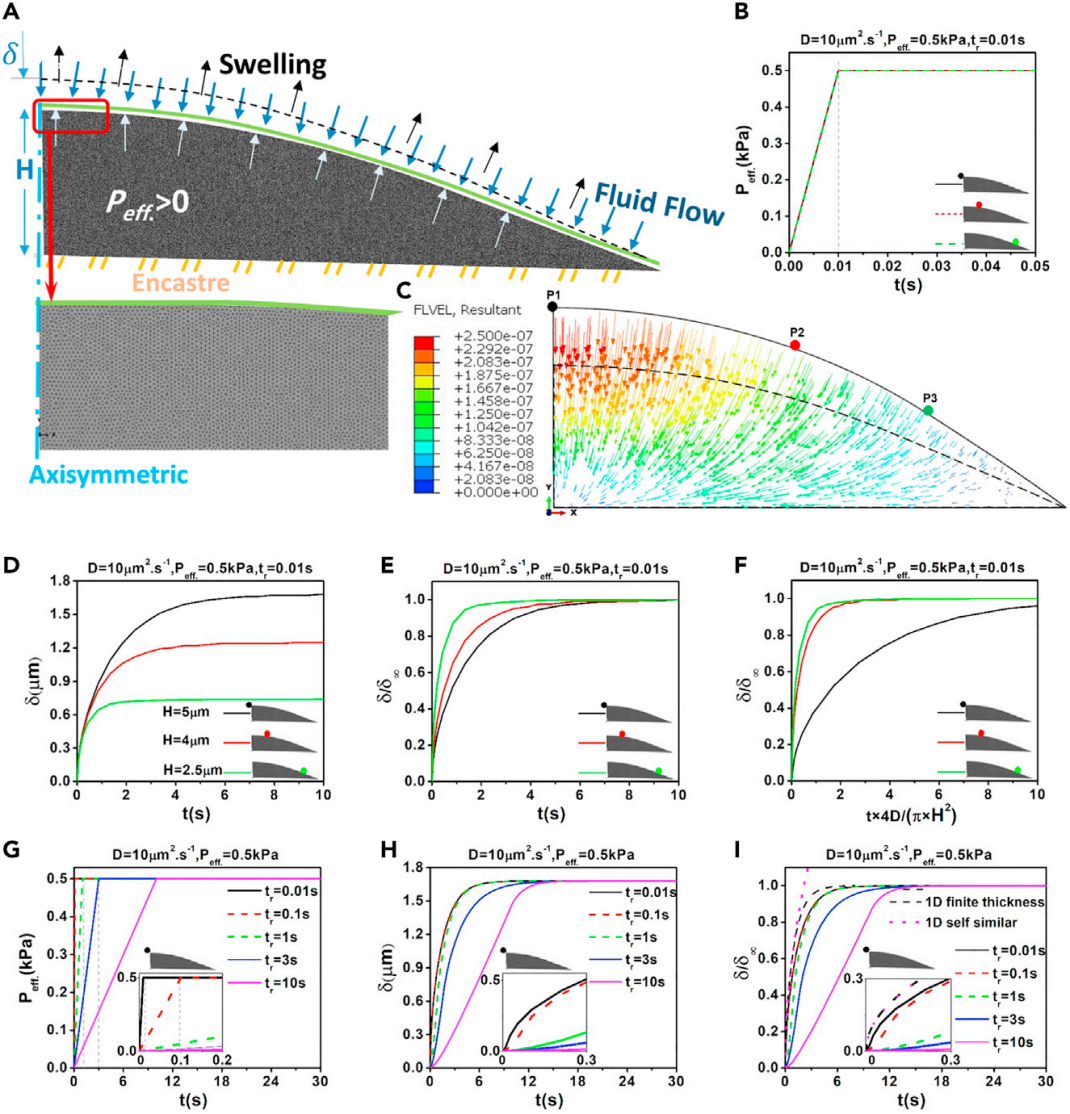
Effects of spatial location, rise time, effective pressure for constrained swelling of a realistic cellular geometry investigated by FE porohyperelastic simulations (A) An axisymmetric FE model of a cell for simulating effective pressure-driven porohyperelastic swelling effectively representing the osmotically driven cell swelling. Geometry (H = 5.5μm and L = 15μm) and boundary conditions of the meshed geometry is shown. (B) Following the application of a change in effective pressure *P*_*eff.*_> 0 → *P*_*in*_ < *P*_*out*_ over rise time t_r_ = 0.01 s, the vertical displacements at positions with different heights (P1, P2 and P3) were computed. (C) The cell swells due to application of pressure changes across cell membrane and entering of water into cell interior. Dash and solid lines indicate non-deformed and deformed shapes respectively. The fluid velocity field is also shown with color arrows. (D) Plots of vertical displacements δ over time considering the pressure change in (C). (E) Normalization of vertical displacements with respect to maximum final vertical displacements $\delta_{\infty }$. (F) Normalization of both vertical displacements (similar to (e)) and time scales using $\tau =\frac{4Dt}{\pi H^{2}}$. (G) Application of a same pressure change but over different rise times t_r_ = 0.01,0.1,1,3,10 s. (H) Plots of vertical displacement at P1 in response to the pressurization conditions from (G). (I) Normalization of the y axis of the curves in (H) using $\frac{\delta_{\;}}{\delta_{\infty \;}}$ and plots of analytical solutions.

Our osmotic perturbation experiments indicate that cells undergo large deformations (strains over 50%) that may fall outside of the linear elasticity theory implemented in FE simulations. Also, our porohyperelastic simulations for a simple geometry (Figure S4) suggest trends that were consistent with the linear poroelastic results (Figure 3). Therefore, we considered a porohyperelastic formulation (Table S2) for an axisymmetric geometry of a typical cell adhered to a substrate and refined the model by employing an unstructured mesh (Figure 4A). Osmotic perturbations were simulated by imposing a linear ramp (*P*_eff._ = 50 Pa) over a fast rise time *t*_*r*_ = 0.01 s (Figure 4B), leading to water influx and a complex velocity field as illustrated in Figure 4C (and Figure S11B for deswelling). The vertical displacement vs time curves (*δ*-*t*) in specific cellular locations (P1, P2, and P3 with respective heights H = 5.5, 4, 2.5 μm) are shown in Figure 4D. Normalization of vertical displacement using $\frac{\delta_{\;}}{\delta_{\infty \;}}$ did not show any meaningful overlapping of the curves. However, normalization of both vertical displacement and time (using $\tau =\frac{4Dt}{\pi H^{2}}$) resulted in some fair overlapping of curves for positions P1 and P2 (Figure 4F). Furthermore, while self-similar (Equation 26) and 1D finite thickness (Equation 25) solutions did not fit the overall trend of FE curves, the self-similar solution shows a good fit for curves at short time scales (Figure S7). Interestingly, for positions with larger heights (e.g. P1 and P2) the self-similar solution appropriately fit the $\frac{\delta_{\;}}{\delta_{\infty \;}}$ FE curves up to 1s (Figures S7A and S7B) while for P3 (that has a significantly lower height), the self-similar solution fit the FE curve only up to 0.5s (Figure S7C). Consistent with our observations in the rectangular geometry (Figure 3), the differences in the dynamics of different locations is due to geometrical boundary effects: in particular, P1 lays on the symmetry axis, and relatively far from the bottom boundaries, while P3 is the farthest of the three points and closest to the bottom.

While our analytical model could capture the dynamics of complex cellular swelling/deswelling at short timescales, it assumes that osmotic perturbations are applied infinitely fast, which does not occur in both *in vitro* experiments and *in vivo* settings. The experimental conditions are more realistically modeled in our FE simulations by considering the effect of transiently rising pressure. Focusing on the P1 position and considering a pressure ramp of 0.5 kPa occurring over *t*_*r*_ = 0.01, 0.1, 1, 3, 10 s (Figure 4G), the *δ*-t curves are plotted in Figure 4H. Normalization of vertical displacement using $\frac{\delta_{\;}}{\delta_{\infty \;}}$ led to the overlap of the curves only when *t*_r_ <<1s and 1D analytical solutions could fit these curves at short time scales. However, for *t*_r_>>1s (i.e. *t*_r_ = 3 and 10s), the shape of the curves significantly differs, yet reaching a plateau of $\delta_{\infty }$ ∼1.65 μm at *t*_*∞*_∼11s (Figures 4H and 4I), suggesting that at the steady-state the poroelastic relaxation responses converge to similar values.

### Experimental validation of the poroelastic model

To consider the effect of transiently changing osmotic pressure in realistic geometries, and obtain the corresponding values of poroelastic parameters, we modeled the living cell as an isotropic porohyperelastic continuum (as described in the previous section), and extracted the material parameters by fitting experimental curves in Figures 2B and 2C with FE simulations. Considering the swelling/deswelling curves, fundamentally we can only extract three parameters (P_eff._, D and t_r_). Therefore, we used the experimental values of elastic modulus estimated from AFM indentation tests (average of swelled and control, Figure 2G, E = 0.975kPa, for swelling and average of shrunk and control, Figure 2G, E = 1.15kPa, for deswelling) and ran optimization procedures to fit the swelling/deswelling experimental curves (Figures 2B and 2C) with FE simulations and estimate effective pressure (*P*_eff._), rise time (*t*_r_) and poroelastic diffusion constant (*D*) for the swelling and deswelling of the HeLa cells. For simplicity, we considered a fixed Poisson's ratio of *υ* = 0.3 as suggested in recent studies (Moeendarbary et al., 2013; Wu et al., 2018; Mokbel et al., 2020). To avoid the influence of active remodeling and intrinsic viscoelasticity of the cytoskeleton, we only considered the first 40 s of swelling/deswelling curves in fitting procedures. The optimization procedure involved initial running of the simulations to fit the experimental curves and find the first approximated values for Peff. and D, considering t_r_ = 0. Then a step increase of 0.1s and small variations in P_eff._ and D were allowed to find the best parameters that fit the experimental curves with a minimal error.

**Figure 5:**
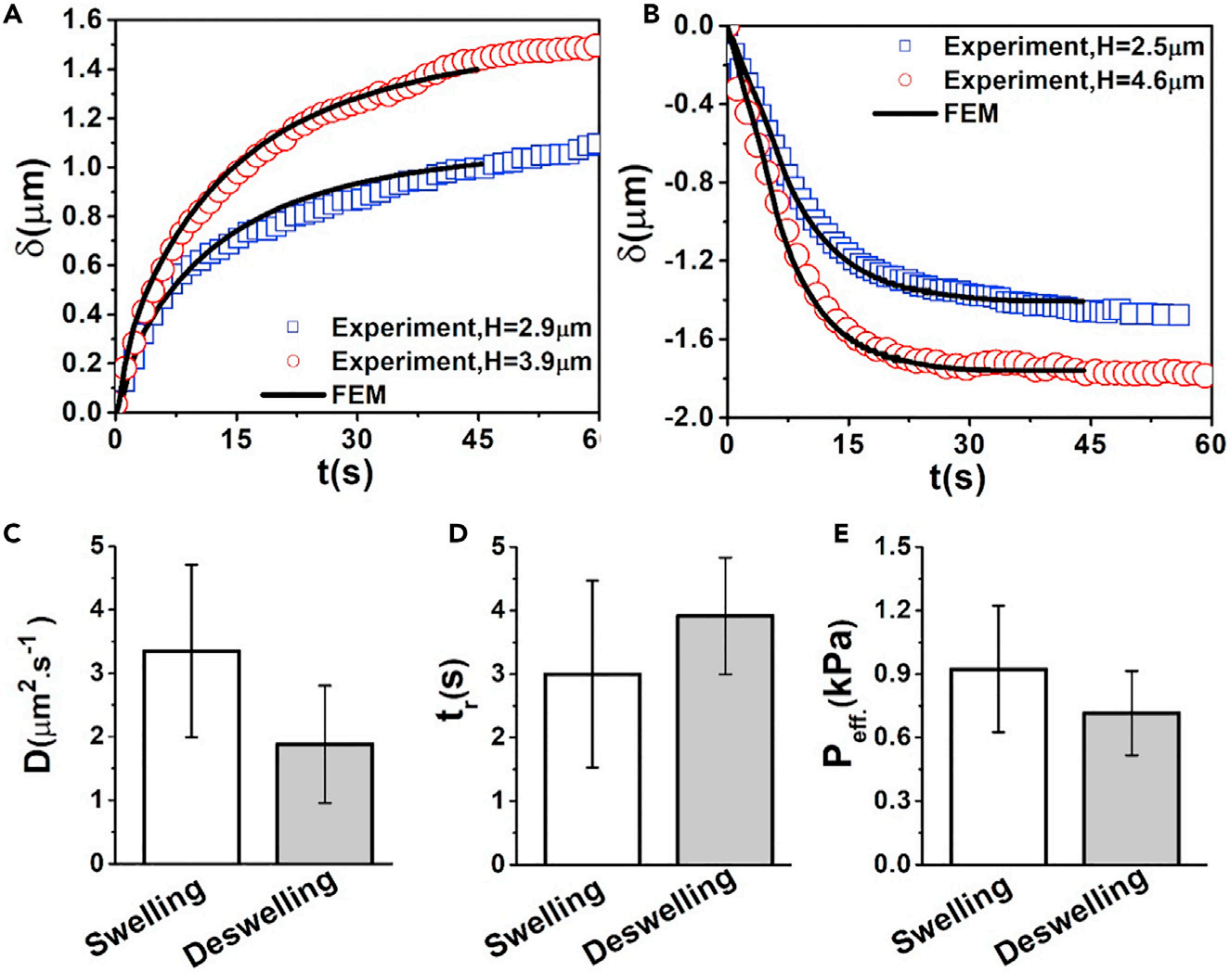
Experimental estimation of cellular poroelastic parameters (A and B) Examples of experimental osmotic responses of HeLa cells fitted against FE simulations for two cases of swelling in (A) and two deswelling cases in (B). (C–E) The average diffusion coefficient, rise time, and effective pressure obtained from fitting of 10 swelling and deswelling experiments with FE simulations. Error bars indicate the standard deviation in all graphs.

Representative results of fitting for two swelling cases of two different cells (with nanobeads positioned at heights of 2.9 and 3.9 μm) and two deswelling cells (with nanobeads positioned at heights of 2.5 and 4.6 μm) are shown in Figures 5A and 5B. We found the following optimum effective pressures, diffusion constants and rise times for swelling: *P*_eff._ = 0.6 kPa, *D* = 2 μm^2^s^−1^ and *t*_*r*_ = 4.5 s for H = 2.9 μm, and *P*_eff._ = 0.8 kPa, *D* = 2.4 μm^2^s^−1^ and *t*_*r*_ = 4 s for H = 3.9 μm (Figure 5A). For deswelling, we kept the same geometry but forced a negative effective pressure and found the following optimum parameters: *P*_eff._ = -0.7 kPa, *D* = 0.8 μm^2^s^−1^ and *t*_*r*_ = 5 s for H = 2.5 μm, and *P*_eff._ = -0.8 kPa, *D* = 3 μm^2^.s^−1^ and *t*_*r*_ = 2.5 s for H = 4.6 μm (Figure 5B). Finally, running the fitting for all experimental curves, we estimated the average *P*_eff._ = 0.93 ± 0.3 kPa, *D*_ave_. = 3.35 ± 1.36 μm^2^.s^−1^ and *t*_*r*_ = 3 ± 1.47 s for swelling and *P*_eff._ = -0.72 ± 0.21 kPa, *D*_ave_. = 1.88 ± 0.92 μm^2^s^−1^, *t*_*r*_ = 3.92 ± 0.92 s for deswelling (Figures 5C, 5D, and 5E).

In our simulations, we only considered the influence of the cell geometrical features and therefore the abovementioned coefficients can only be regarded as approximations to the bulk, average poroelastic properties of a living cell. The structural heterogeneity and complex nature of the cell can be the key reason that different experimental settings report different values for cellular mechanical and rheological properties. Indeed, cellular structures such as the cell membrane, the cortex, the nucleus, and the granular filamentous interior, with different permeability and elasticity characteristics, contribute to setting the overall swelling/shrinking cell dynamics.

## Discussion

AFM indentation test results in Figures 2F and 2G show that cells exposed to sucrose and after deswelling exhibited no significant changes in elastic modulus or diffusion constant. Nonetheless, the mild increase in cell elastic modulus (∼10%), although not statistically significant, is comparable in magnitude to the stiffening of cytoplasmic material after water efflux (Guo et al., 2017) and indicates that cells relax less rapidly and become stiffer with decreasing fluid fraction. Consistent with previous AFM indentation results by us and others, we measured the diffusion coefficients in the range from *D* = 10 to 20 μm^2^.s^−1^ (Charras et al., 2005, 2009; Moeendarbary et al., 2013) and elastic modulus of *E ∼* 1 kPa (Lim et al., 2006; Moeendarbary et al., 2013; Gautier et al., 2015). Also, increases in cell volume resulted in a significant increase in poroelastic diffusion constant and a significant decrease in cellular elastic modulus. Our indentation simulations on the final swelled/shrunk state of a simple geometry (see STAR Methods) showed that *D* increased while *E* decreased for the swelled state, and reversely *D* decreased and *E* increased for the shrunk state (Figures S12 and S13) which are consistent with our experimental observations.

Within the timescale of our experiments, and separating swelling and shrinkage phenomena, a unique set of poroelastic parameters reproduced most of the features of fast volume changes in HeLa cells. This is important as it provides the ability to better understand the early adaptive physiological responses of living cells to external osmotic challenges. However, the diffusion coefficients estimated from experimental swelling/deswelling curves are around one order of magnitude smaller than those obtained from AFM indentation tests. Several reasons could contribute to this discrepancy. Firstly, in AFM tests, a thin layer of actin cortex located beneath the plasma membrane mostly contributes to the measured poroelastic parameters. During the osmotic perturbation, however, water infiltration through the whole cell, including the cytoplasm and specific regions of the cell such as cell nucleus, regulate the dynamics of swelling/deswelling (Potma et al., 2001). Furthermore, while in AFM tests the pressurized fluid redistributes only horizontally in small sections of the membrane because of the compression of the impermeable spherical indenter, the whole-cell membrane significantly controls the osmoregulated volume changes in swelling/deswelling experiments. Finally, the timescale of ramp (pressurization) and relaxation phases are different across the two methodologies. In the AFM experiments, we used t_r_∼500ms while, in osmotic tests, it takes a certain amount of time for the osmotic perturbations to act on cell surfaces (t_r_ in the order of a few seconds) and delays associated with media mixing could be another reason for the discrepancy in the estimation of diffusion coefficients.

Comparing deswelling and swelling experiments, the consistently faster poroelastic diffusion in deswelling might be due to the irreversible dynamics of membrane water channels (aquaporins) (Mitchison et al., 2008; Ibata et al., 2011) and to the variable responses of the cytoskeleton (and indeed whole-cell components) and to tensional and compressional forces created during hyperosmotic shrinkage and hypoosmotic swelling (Sehy et al., 2002; Mitchison et al., 2008). The imaging of actin cytoskeleton (which is the main determinant of cellular mechanical properties) before and after short-time of application of osmotic perturbations (Figures 2D and 2E), suggests that while cytoskeleton can be considered elastic at very short timescales (as remodeling and reorganization of actin cytoskeleton occurs at long time scales), slight variations of cytoplasmic pore size and crowding can effectively influence the poroelastic properties at short timescales. Although the intrinsic viscoelastic timescale of the cell cytoskeleton may fall within a range close to poroelastic timescales, the early osmoregulation of the cell volume is predominantly regulated by fluid-solid interactions, that is, the poroelasticity.

Accurate predictions of cell volume changes in response to pressure challenges can help to quantify the mechanical behavior of living cells and contributes to the understanding and diagnosis of various pathologies (Liu et al., 2019a, 2019b). More importantly, such a predictive model can help to accelerate biomedical drug discovery and clinical research into novel cancer treatments, as well as advancing other fields such as bioengineering and tissue regeneration, drug delivery, hygiene products, and microfluidic organ-on-chip technologies (Borsali and Pecora, 2008; Feksa et al., 2018; Whelan et al., 2021). In the present work, we used the finite element method to simulate the osmotic-driven swelling and deswelling kinetics of the cytoplasm of a living cell. In cell biology, it is important to be able to model and predict the response of different cell types to osmotic perturbations using models that incorporate realistic time frames for physiological phenomena. In the context of tumor biology, for example, being able to predict the response of cancer cells to chemotherapies that alter their internal hydrostatic pressure may help to explain their effectiveness at treating tumors, or conversely, may reveal how tumor cells develop resistance to a particular drug (Micalet et al., 2021). Our model closely recapitulates the volumetric responses of HeLa cells with rapid redistribution of fluid due to hypo and hyperosmotic stimuli. We confirm that the poroelastic model, with minimal parameters, explains the swelling/deswelling of living cells *in vitro* and found that, in both simplified and cell-like geometries, the effect of duration of initial stimulation, that is, rise time, is critical. We also found that including more realistic geometries obtained from confocal reconstructions images of the cell improves uncertainties arising from spatial inhomogeneities and is simply sufficient for predicting the cell mechanical responses and volume regulation without the need for additional complex parameters.

### Limitations of study

The main limitation of our work is the consideration of isotropy and constant values for poroelastic parameters. However, both simulations (Figures S12 andS13) and experiments (Figures 2F and 2G) on swelled and shrunken cell states suggest that the effective elastic modulus and diffusion constants progressively change during volume changes. Interestingly, these changes are in the opposite direction for swelling compared to deswelling and this may explain the experimentally observed differences between the dynamics of swelling and deswelling. Also, additional features, such as the viscoelasticity of the solid-phase, were not considered in our model. In swelling experiments, most vertical displacements were larger for greater heights while this trend was weaker in deswelling cases. Therefore, we speculate that in the deswelling experiments, the height dynamics may strongly be affected by the position of the nucleus and the presence of the (stiffer) nucleus may have a stronger impact during deswelling compared to swelling. Notably, in this study we did not consider the presence of the nucleus and, therefore, the inclusion of the nucleus and its properties would be an interesting addition in future studies.

## STAR★Methods

### Key resources table

**Table:** 

REAGENT or RESOURCE	SOURCE	IDENTIFIER
**Chemicals, Peptides, and Recombinant Proteins**		

10% Fetal Bovine Serum	Gibco Life Technologies, Paisley, UK	A3160501
DMEM culture medium	Gibco Life Technologies, Paisley, UK	11054001
1% Penicillin-Streptomycin	Gibco Life Technologies, Paisley, UK	15140122
Hoechst 34332	Merck-Biosciences	62249
NPPB	Tocris, Bristol, UK	0593
DCPIB	Tocris, Bristol, UK	1540
500 nm Carboxylated green-yellow beads	Molecular Probes, Invitrogen	FluoSpheres, F8812

**Experimental models: Cell lines**		

Life-Act ruby HeLa cells	Laboratory of Guillaume Charras	https://doi.org/10.1038/NMAT3517

**Software and algorithms**		

ABAQUS	Dassault Systèmes	v 2018
Origin	OriginLab	2018 (b9.5.193)
ImageJ	National Institude of Health, USA	v 1.52i
Matlab	MathWorks	2017a (9.2.0.538062)
μManager	Micromanager, Palo-Alto, CA	

**Other**		

Atomic force microscope	JPK, Instruments, Germany)	JPK Nanowizard-I
Spinning disk confocal microscope	Yokogawa, Japan	CSU-22
Piezo-electric z-drive	NanoscanZ	Prior Scientific, Rockland
Confocal laser scanning microscope	Olympus, Japan	FV1000
50 mm glass bottomed Petri dishes	World Precision Instruments, Milton Keynes, UK	Fluorodish

### Resource availability

#### Lead contact

Further information requests should be directed to the lead contact, Professor Emad Moeendarbary, (e.moeendarbary@ucl.ac.uk).

#### Materials availability

This study did not generate new reagents.

### Experimental model and subject details

#### Cell culture

HeLa cells expressing cytoplasmic GFP or Lifeact-Ruby were cultured at 37°C and 5% CO_2_ in DMEM culture medium (Gibco Life Technologies, Paisley, UK) supplemented with 10% Fetal Bovine Serum (FBS, Gibco Life Technologies) and 1% Penicillin-Streptomycin (Gibco Life Technologies, Paisley, UK).

### Method details

#### Cell volume measurements

Confocal stacks of cells expressing cytoplasmic GFP were acquired using a spinning disk confocal microscope (Yokogawa CSU-22, Japan, 100x oil immersion Olympus objective lens, NA = 1.4, piezo-electric z-drive, NanoscanZ, Prior Scientific, Rockland) to measure changes in cell volume in response to osmotic shock. Exposure time and laser intensity were set to minimize photobleaching and stacks (40 images separated by 0.2 μm) were acquired for a total of 30 mins with the shortest achievable time interval (∼2 min).

Using Matlab (Mathworks Inc) Image Processing Toolbox, the background noise of stack images was removed, and images were smoothed and binarised. A series of erosion and dilatation operations were performed on binarised stacks to create a contiguous cell volume image devoid of isolated pixels (Figure 1A). The cell volume at each time step was calculated from the sum of nonzero pixels in each stack multiplied by the volume of a voxel. Prior to application of osmotic shock, four stacks were captured and then cell volume was followed for a further 25 minutes (Figure 1B) after the change in osmolarity. For hyperosmotic shocks, cells were exposed to medium to which 100mM and 250mM sucrose had been added. For hypoosmotic conditions, we diluted culture medium using water and observed a transient swelling, signifying that regulatory volume decrease mechanisms acted on the time-scale of the experiment. Therefore, cells were treated with regulatory volume decrease inhibitors (Lang et al., 1998) NPPB and DCPIB (N+D on the graph) to ensure stable volume increases over the longer time-scale necessary for AFM measurements (∼30 min). The measured volume at each time point was normalized to the initial cell volume at t=0 s (Figure 1B). Additionally, to investigate effects of osmotic perturbations on cellular structures, a confocal laser scanning microscope (FV1000, Olympus) was used to image F-actin using the live reporter construct Lifeact-Ruby (Figures 2D and 2E) and the nucleus using the cell-permeant nuclear marker Hoechst 34332 (Merck-Biosciences).

#### Attachment of nanobeads to the cell surface and measurement of cell height

HeLa cells were plated onto 50 mm glass bottomed Petri dishes (Fluorodish, World Precision Instruments, Milton Keynes, UK). Yellow-Green carboxylate-modified fluorescent nanobeads with a diameter of 500 nm (FluoSpheres, Molecular Probes, Invitrogen) were coated with collagen-I following the manufacturer's protocol. To attach nanobeads to the cell membrane prior to experiments, Life-Act ruby HeLa cells were incubated for ∼30 min with a diluted solution containing the fluorescent collagen coated nanobeads. A Piezo-electric z-stage (NanoscanZ, Prior, Scientific, Rockland, MA) was used to calibrate defocused images and to estimate the height of the attached nanobeads (Figures 1C and 1D). The z-stage was fixed on top of the optical fluorescence microscope stage (IX-71, Olympus) and was piloted using μManager (Micromanager, Palo-Alto, CA) to step up/down while taking epifluorescence images. In order to find the relationship between the z-positions of the bead relative to the plane of focus and the radius of the outer rings formed in the defocused images, we acquired a series of images of the nanobead for different distances from the coverslip with step size of 100 nm. The initial height of the bead (vertical distance between the bottom of cell attached to the coverslip to the bead located on cell membrane) is a critical parameter for estimating the poroelastic diffusion constant using either analytical solutions or FE simulations. The position of the bead relative to the coverslip was estimated by acquiring fluorescence z-stack images of the cells and beads attached to cell with a step size of 200 nm over a distances of ∼10 μm. Starting a few microns above the cell and ending a few microns below the bottom of the glass coverslip, the z-planes that showed the stress fibres at the bottom of the cell in focus were considered as the bottom of cell (the reference zero height), and the plane in which the bead was in focus was considered as the location of bead and therefore the initial height of the bead was estimated (Figure 1C).

#### Principle of defocusing microscopy

Through tracking of fluorescent nanobeads attached to the cell membrane (Figure 1C), we monitored the response of cells under different hyperosmotic or hypoosmotic perturbations and investigated the swelling/deswelling kinetics of the cytoplasm. Imaging away from the nanobead plane, the bead appears as a set of concentric rings in the image plane (Figure 1D) and the shape and size of the rings depends on the diffraction pattern of the bead and point spread function of the imaging system (Rosenbluth et al., 2008). Off-plane defocused images of fluorescent nanobeads (Figure 1D) enabled precise measurements of time-dependent cellular deformations in the z-direction with ∼10 nm resolution (Perlman and Bhattacharya, 2007; Rosenbluth et al., 2008; Kang et al., 2009; Yoon et al., 2010; Bottier et al., 2011; Moeendarbary et al., 2013).Furthermore, the defocused images of the nanobead were taken at 100 ms intervals providing an excellent temporal tracking resolution. The spatio-temporal resolution of defocusing technique is significantly higher than spinning disk confocal microscopy enabling precise investigation of cell volume changes at short timescales. We focused on a certain plane that shows a slightly defocused image of the bead and while acquiring images (at 100 ms intervals), either a hypoosmotic or a hyperosmotic shock were applied to drive water in (swelling) or out (deswelling) of the cell, respectively. After application of such osmotic pressure gradients, changes in the positions of the membrane-bound fluorescent nanobeads over time were determined by monitoring the evolution of the radius of the ring in the defocused fluorescent images (Figures 1D, 1E, and 1F).

#### Image processing to determine changes in cell height from radial projection

A 2D defocused image of a bead is circularly symmetric, thus a 1D radius function was used to detect the radius of the outer ring in each defocused image. Because there were several nanobeads attached to each cell, the rough central position of the bead of interest was first selected visually and then the precise coordinates of the centroid of the chosen bead $\left(x_{c},y_{c}\right)$ were estimated by fitting a 2D Gaussian function to the image. An arbitrary annulus interval Δ was set to generate a radial distance vector $r_{i}=i\Delta ,\;i=0\ldots N$. By knowing the precise center of the image, the distance of each pixel in the image from the centroid was estimated. According to the radial distance of each pixel *d*, there were a total of $P_{i}$ pixels lying between radii $r_{i-1}$ and $r_{i+1}$. For each pixel within the annulus between radii $r_{i-1}$ and $r_{i+1}$ the averaged radial intensity vector ${\text{I}}_{R}\left(r_{i}\right)$ was calculated (see Figure 1E) by splitting the intensity of each pixel *I* in this annulus linearly according to its radial position *d*:

$${\text{I}}_{R}\left(r_{i}\right)=\frac{1}{\sum_{n=1}^{P_{i}}w_{n}}\overset{P_{i}}{\underset{n=1}{\sum }}w_{n}I_{n}$$

(Equation 1) $$w_{n}=\left\{ \begin{array}{l} \frac{d-r_{i-1}}{\Delta }\;if\;r_{i-1}\leq d<r_{i}\;\\ \frac{r_{i+1}-d}{\Delta }if\;r_{i}\leq d<r_{i+1} \end{array}\right.$$

To find an estimate with sub-pixel resolution for the radial position of the outer rings, the averaged radial intensity curve was normalized to its maximum intensity as shown in the inset of Figure 1E. The intersection of the curve with the line y = 0.05 provided a good estimate for the radial position where the intensity of the outer ring diminishes to zero. The changes in the height of cells versus time were obtained after hypo/hyperosmotic shock were normalized with respect to the final change in cell height. The curves were then used for fitting with analytical solutions and for comparison to FE simulations.

#### Atomic force microscopy measurements

To estimate the cellular poroelastic parameters including the elastic modulus and poroelastic diffusion constant, we employed atomic force microscopy (AFM) indentation tests (JPK Nanowizard-I, JPK, Instruments, Germany) on central regions of the cells adhered on glass bottom petri dishes. The AFM cantilever (MLCT, Bruker, interfaced with 15 μm latex microspheres, Invitrogen) was pressed into the cells with a fast approach velocity of 10 μm s^−1^ to excite poroelastic modes of relaxation. Once a maximum force of 5 nN was reached, the cantilever was kept at constant height (via z-closed loop feedback implemented on the JPK Nanowizard) for 5s to enable acquisition of the force-relaxation curves. The force-indentation and force-relaxation curves were analysed to extract the elastic modulus and poroelastic diffusion coefficient (see (Moeendarbary et al., 2013) for detailed methodology and curve fitting procedures).

#### One-dimensional analytical solution

To study the poroelastic behaviors of living cells, we first evaluated the applicability of one dimensional analytical solution to investigate the swelling/shrinkage kinetics of simple 2D geometries under the framework of linear poroelasticity. We considered the swelling/deswelling kinetics of a linearly isotropic poroelastic material fully immersed in a solvent and bound to an impermeable substrate on its bottom surface (Figures 3A and S1). Consider a thin layer of a poroelastic material attached from one side (Figure S1) to a fixed substrate and immersed in a solvent for a long enough time to reach an equilibrium, that is, a state of homogeneity where C_0_ and μ_0_ are the initial concentration and chemical potential of solvent respectively. In the present study, the initial state is assumed to be isotropically swollen from the dry state (Yoon et al., 2010; Yang et al., 2014; Tien et al., 2015; Malandrino and Moeendarbary, 2019). We consider the quasistatic deformation of an isotropic fully saturated poroelastic medium with a constant porosity. The constitutive equation is an extension of linear elasticity to poroelastic materials first introduced by Biot (Biot, 1941). Biot's formulation can be simplified when poroelastic parameters assume their limiting values. Under the incompressible condition, undrained bulk modulus is much larger than drained bulk modulus. Also for soft materials, the value of Biot coefficient is considered to be one and the constitutive equation relates the total stress tensor σ to the infinitesimal strain tensor ε of the solid phase and the pore fluid pressure *p* (Yoon et al., 2010; Yang et al., 2014; Tien et al., 2015; Malandrino and Moeendarbary, 2019):

(Equation 2) $$\sigma =2{\text{G}}_{S}\varepsilon +\frac{2{\text{G}}_{S}{\text{ν}}_{S}}{\left(1-2{\text{ν}}_{S}\right)}tr\left(\varepsilon \right)I-pI$$

where ${\text{G}}_{\text{s}}$ and $\nu_{\text{s}}$ are the shear modulus and the Poisson ratio of the drained network respectively, and θ=tr ε the variation in fluid content. This equation is similar to the constitutive governing equation for conventional single phase linear elastic materials. However the time dependent properties are incorporated through the pressure term that acts as an additional external force on the solid phase. In the absence of body forces and neglecting the inertial terms, the local stress balance results in the equilibrium equation (Cai et al., 2010; Yoon et al., 2010; Malandrino and Moeendarbary, 2019)

(Equation 3) div σ+F=0→div σ=0

Darcy's law considers fluid transport through the non-deformable porous medium

(Equation 4) q=−K∇p

where q is the filtration velocity and *K* the hydraulic permeability. Neglecting any source density, the mass conservation of a fluid yields:

(Equation 5) $$\text{div }q=-\frac{\partial \text{θ}}{\partial \text{t}}$$

Combining the continuity equation and Darcy's law results in (Malandrino and Moeendarbary, 2019)

(Equation 6) $$\frac{\partial \text{θ}}{\partial \text{t}}=\text{K}\nabla^{2}p$$

Applying the equilibrium condition to the constitutive law, one can obtain the Navier equations

(Equation 7) $${\text{G}}_{\text{S}}\nabla^{2}u+\frac{{\text{G}}_{\text{S}}}{\left(1-2{\text{ν}}_{\text{S}}\right)}\nabla \text{ div }u-\nabla \text{p}=0$$

The fundamental poroelastic equations for swelling/shrinkage of gels under osmotic perturbation are very similar with a redefinition of some parameters such as pressure and fluid flux at boundaries. Let us consider the initial state of the gel to be homogeneous and free from mechanical load with C_0_ the initial concentration of solvent inside the gel and ${\text{μ}}_{0}$ the initial chemical potential of the gel. Equation (Equation 5) rewritten in terms of the concentration of the solvent in the gel is:

(Equation 8) $$\text{div }q=-\frac{\partial C}{\partial \text{t}}$$

Darcy's law (Equation 4) defines the migration of the solvent in the gel, thus this equation transforms to:

(Equation 9) $$q=-\left[\frac{\text{K}}{{\text{Ω}}^{2}}\right]\nabla \text{μ}$$

Where Ω is the volume per solvent molecule and μ the time-dependent chemical potential of the solvent. Incompressibility of the network and the solvent imply that the change in volume of the gel is only due to migration of solvent molecules into or out of the network which changes the volume of solvent inside the gel:

(Equation 10) $$tr\;\varepsilon =\text{Ω}\left(C-C_{0}\right)$$

At thermodynamic equilibrium, the work done on each element of the gel equals the change in free energy δW written as:

(Equation 11) $$\delta W=tr\left(\sigma .\delta \varepsilon \right)+\left(\text{μ}-{\text{μ}}_{0}\right)\left(\delta C\right)$$

where W is the Helmholtz free energy per unit volume of the gel. In this relationship tr(σ.δε) is the mechanical work due to stress and $\left(\text{μ}-{\text{μ}}_{0}\right)\left(\delta C\right)$ is the work done by the chemical potential. Using equation (Equation 9) one can write the change in free energy in terms of only the strain tensor $\delta W=tr\left(\sigma .\delta \varepsilon \right)+\left(\text{μ}-{\text{μ}}_{0}\right)\left(tr\;\varepsilon /\text{Ω}\right)$ which yields:

(Equation 12) $$\sigma =\frac{\partial W\left(\varepsilon \right)}{\partial \varepsilon }-\frac{\left(\text{μ}-{\text{μ}}_{0}\right)}{\text{Ω}}I$$

The free energy can be written as a function of the strain tensor in the linear case of an isotropic gel:

(Equation 13) $$W=G_{s}\left[tr\left(\varepsilon^{2}\right)+\frac{\upsilon_{S}}{1-\upsilon_{S}}tr\left(\varepsilon^{2}\right)\right]$$

Combining equations (Equations 12 and 13) results in equation (Equation 2) replacing pore pressure by the term $\left(\text{μ}-{\text{μ}}_{0}\right)/\text{Ω}$:

(Equation 14) $$\sigma =2G_{s}\varepsilon +\frac{2G_{s}\upsilon_{S}}{\left(1-2\upsilon_{S}\right)}tr\left(\varepsilon \right)I-\frac{\left(\text{μ}-{\text{μ}}_{0}\right)}{\text{Ω}}I$$

Navier and diffusion equations similar to the Biot poroelasticity equations can then be derived using the above equations:

(Equation 15) $$G_{s}\nabla^{2}u+\frac{G_{s}}{\left(1-2\upsilon_{S}\right)}\nabla \text{ div }u-\frac{1}{\text{Ω}}\nabla \text{μ}=0$$

(Equation 16) $$\frac{\partial \text{C}}{\partial \text{t}}=D\nabla^{2}\text{C}$$

These equations can be used to define the dynamics of gel swelling/shrinkage in response to a change in extracellular osmolarity and to extract diffusion coeffcient *D*. Here we consider the situation where all the components of the strain tensor except ${\text{ε}}_{\text{zz}}=\frac{\partial {\text{u}}_{\text{z}}}{\partial \text{z}}$ are zero so that the poroelastic material is only allowed to deform in one direction (Figure S1). In this case the non-zero components of the stress tensor reduce to:

(Equation 17) $$\left\{ \begin{array}{l} \sigma_{zz}=\frac{2G_{s}\left(1-\nu_{s}\right)}{\left(1-2\nu_{s}\right)}\varepsilon_{zz}-p\\ \sigma_{yy}=\sigma_{xx}=\frac{2G_{s}\nu_{s}}{\left(1-2\nu_{s}\right)}\varepsilon_{zz}-p=\frac{\nu_{s}}{\left(1-\nu_{s}\right)}\sigma_{zz}-\frac{\left(1-2\nu_{s}\right)}{\left(1-\nu_{s}\right)}p \end{array}\right.$$

Substituting ${\text{u}}_{\text{z}}$ as the only component of the displacement field into the Navier equation (Equation 7), we have

(Equation 18) $$\frac{2G_{s}\left(1-\nu_{s}\right)}{\left(1-2\nu_{s}\right)}\frac{\partial^{2}u_{z}}{\partial z^{2}}-\frac{\partial p}{\partial z}=0$$

Combining equations (Equations 6 and 17) and using the fact that ${\text{ε}}_{\text{zz}}=\text{θ}$, the diffusion equation for the pore pressure is obtained:

(Equation 19) $$\frac{\partial p}{\partial t}-D\frac{\partial^{2}p}{\partial z^{2}}=-\frac{\partial \sigma_{zz}}{\partial t}$$

This equation is an inhomogeneous diffusion equation that can be solved by specifying a stress condition. In the following the one dimensional time-dependent settlement of a poroelastic material subjected to certain types of boundary conditions is studied. Consider a thin layer of a poroelastic material (a gel) attached from one side (Figure S1) to a fix substrate and immersed in a solvent for a long enough time to reach an equilibrium homogeneous state with C_0_ and ${\text{μ}}_{0}$ the initial concentration and chemical potential of solvent respectively. When a solvent with different chemical potential of $\bar{\text{μ}}$ is suddenly exposed to the gel, the chemical potential gradient $\bar{\text{μ}}-{\text{μ}}_{0}$ drives the solvent to flow into/out of the gel (Yoon et al., 2010). The displacement and chemical potential fields can be considered one dimensional in *z* if the lateral dimensions of the gel are much larger than its thickness (${\text{L}}_{\text{x}},{\text{L}}_{\text{y}}\gg \text{H}$) then there is a negligible amount of flow from the edges and the solvent flows mostly from top surface. The gel is constrained in *x-y* so ${\text{ε}}_{\text{xx}}={\text{ε}}_{\text{yy}}=0$. The gel can freely swell and deswell from the top surface (${\text{σ}}_{\text{zz}}=0$) (Yoon et al., 2010; Bouklas and Huang, 2012) and thus the equations (Equations 17 and 19) can be written in terms of chemical potential:

(Equation 20) $$\left\{ \begin{array}{l} \varepsilon_{zz}=\frac{\partial u_{z}}{\partial z}=\frac{\left(1-2\nu_{s}\right)}{2G_{s}\left(1-\nu_{s}\right)}\frac{\left(\text{μ}-{\text{μ}}_{0}\right)}{\text{Ω}}\\ \sigma_{xx}=\sigma_{yy}=-\frac{\left(1-2\nu_{s}\right)}{\left(1-\nu_{s}\right)}\frac{\left(\text{μ}-{\text{μ}}_{0}\right)}{\text{Ω}} \end{array}\right.$$

(Equation 21) $$\frac{\partial \text{μ}}{\partial t}=D\frac{\partial^{2}\text{μ}}{\partial z^{2}}$$

Considering the top surface as the reference point, the spatial and temporal evolution of chemical potential can be derived using appropriate boundary and initial conditions. The equilibrium chemical potential before application of osmotic shock yields the initial condition $\text{μ}\left(\text{z},0\right)={\text{μ}}_{0}$. Step change in chemical potential on top surface which remains constant over time $\text{μ}\left(0,\text{t}\right)=\bar{\text{μ}}$ and the no flow condition at the bottom ${\frac{\partial \text{μ}}{\partial \text{z}}|}_{\text{z}=-\text{H}}=0$ yields the boundary conditions. Therefore analytically, the solution of the diffusion (Equation 21) is:

(Equation 22) $$\frac{\text{μ}\left(z,t\right)-\bar{\text{μ}}}{{\text{μ}}_{0}-\bar{\text{μ}}}=-\overset{\infty }{\underset{n=1,3,\ldots }{\sum }}\frac{4}{m\pi }\sin\left(\frac{n\pi z}{2H}\right)\text{ exp}\left(-n^{2}\pi^{2}\tau \right)$$

where $\text{τ}=D\text{t}/4{\text{H}}^{2}$ is the dimensionless time (Yoon et al., 2010; Bouklas and Huang, 2012). Now we consider the case where the poroelastic material is infinitely extended in the z direction. In this case, all the boundary and initial conditions are the same as finite domain problem except that ${\text{u}}_{\text{z}}=0$ when z=+∞ which implies that the dissipations arising from solid fluid interactions vanishes to zero at infinity, ∂p/∂z=0 (Yoon et al., 2010; Bouklas and Huang, 2012; Malandrino and Moeendarbary, 2019). The solutions for the normalized chemical potential for infinite poroelastic domain (or early stage solution) can be written as:

(Equation 23) $$\frac{\text{μ}\left(z,t\right)-\bar{\text{μ}}}{{\text{μ}}_{0}-\bar{\text{μ}}}=erfc\left(-\frac{z}{\sqrt{4Dt}}\right)$$

After long enough time $\text{t}\gg {\text{H}}^{2}/\text{D}$ the solvent inside the gel reaches to the equilibrium $\text{μ}=\bar{\text{μ}}$ and gel shrinks/swells from its initial height H by ${\text{δ}}_{\infty }$ to its final height (Yoon et al., 2010). Considering the total final strain at very long times ${\text{ε}}_{\text{zz}}=\frac{{\text{δ}}_{\infty }}{\text{H}}$ and employing equation (Equation 20) the final change in thickness of the gel is:

(Equation 24) $$\delta_{\infty }=\frac{\left(1-2\nu_{s}\right)}{2G_{s}\left(1-\nu_{s}\right)}\frac{\left(\text{μ}-{\text{μ}}_{0}\right)}{\text{Ω}}\text{H}$$

Using equations (Equations 22 and 24) integrating equation (Equation 20) the change of thickness (${\text{u}}_{\text{z}}$ at z=0) is derived as 1D finite thickness solution:

(Equation 25) $$\frac{\delta \left(t\right)}{\delta_{\infty }}=1-\overset{\infty }{\underset{n=1,3,\ldots }{\sum }}\frac{8}{m^{2}\pi^{2}}\exp\left(-n^{2}\pi^{2}\tau \right)$$

Also using equation (Equation 25) the change in the thickness of the gel is written as 1D self-similar solution:

(Equation 26) $$\frac{\delta \left(t\right)}{\delta_{\infty }}=\frac{2}{\text{H}}\sqrt{\frac{Dt}{\pi }}$$

Standard poroelastic solutions were derived by considering only one dominant direction for the material deformation. The displacement and chemical potential fields can be considered one dimensional if the lateral dimensions of the gel are much larger than its thickness and consequently there is a negligible amount of flow from the edges and the solvent flows mostly from the top surface. The gel is constrained to the substrate from its bottom surface and can freely swell and deswell from the top surface. In this case, the non-zero components of the stress tensor reduce to an inhomogeneous pressure diffusion equation that can be analytically solved by specifying appropriate boundary and initial conditions. The initial condition is the equilibrium chemical potential before the application of the osmotic shock and the boundary conditions are the time-independent step change (ramp) of chemical potential at the top surface, and a no-flow condition at the bottom surface. When a solvent with different chemical potential of $\bar{\text{μ}}$ is suddenly exposed to the gel, the chemical potential gradient $\bar{\text{μ}}-\text{μ}=0$ drives the solvent to flow into/out of the gel. While the dimensionless time is defined as $\text{τ}=D\text{t}/4{\text{H}}^{2}$, after long enough time $\text{t}\gg {\text{H}}^{2}/D$ the solvent inside the gel reaches equilibrium $\text{μ}=\bar{\text{μ}}$ and the gel shrinks/swells from its initial height H by *δ* to its final height, *δ*_*∞*_. In the case where the poroelastic material is infinitely extended in the vertical direction (infinite half-space), the dissipations arising from solid-fluid interactions vanish at infinity leading to a self-similar solution (Equation 26), used to obtain the change in the thickness of the gel, *δ(t)/δ*_*∞*_. For the finite height condition, we derived the solution for the change in gel thickness, *δ(t)* (Equation 25), to define the dynamics of a poroelastic gel in response to step pressure changes.

#### Finite element simulations

To model the poroelastic behavior of the cytoplasm, we ran FE simulations of a poroelastic cell that swells or shrinks in response to application of effective pressure changes at its top boundary representing the effects of osmotic changes in the extracellular medium. To investigate the spatial and rise time effects, we first idealised the cell geometry by approximating it to a thin cylindrical disk, with a diameter of 30 μm and a thickness of 5.5 μm, immersed in a solvent sufficiently long to enable it to reach an equilibrium effective pressure with the same value as the outside cell pressure (*P*_*eff.*_ = *P*_out_-*P*_in_ = 0). The net pressure inside (*P*_in_) and outside (*P*_out_) of the cell may arise from different combinations of osmotic and hydrostatic pressures. Both free-sliding (free swelling) and non-slip conditions (constrained swelling) for the bottom surface were studied while a no flow condition was always imposed at the bottom surface (Figure 3A). Later, we considered a more realistic geometry and modeled the cell as an axisymmetric elliptical cap rigidly fixed to a substrate (Figure 4A). The finite element model of both the idealized and realistic cell was constructed in ABAQUS (version 2018). The geometry and boundary conditions of each discretized model are shown in Figures 3A and 4A, respectively. Given the large mechanical deformation range observed in cell biology (Kang et al., 2008; Lin et al., 2008; Ding et al., 2013; Vargas-Pinto et al., 2013; Nguyen et al., 2016; Zhang and Yang, 2017; Castro et al., 2018), and particularly during our osmotic perturbation experiments, we employed nonlinear-geometry (large displacement formulation) and unstructured mesh options in our FE simulations, and also considered a neo-Hookean isotropic porohyperelastic model (large strain formulation) (Lim et al., 2006; Moeendarbary et al., 2013; Vargas-Pinto et al., 2013; Nguyen et al., 2016; Castro et al., 2018; ). The initial shape of HeLa cells was estimated considering 3D stacks of confocal images of a typical cell (Figure 1A). Averaged elastic modulus, estimated from AFM experiments on the swelled and deswelled cells, were considered in the FE simulations to fit the experimental swelling/deswelling curves and obtain P_eff._, D and t_r_. Our linear isotropic homogeneous assumptions provided a good approximation to capture the dynamics of cell volume changes using a small number of independent parameters.

The implicit ABAQUS/standard finite element solver was applied with automatic time steps of Newton-type iterations. A typical swelling/deswelling simulation included a ramp phase (0 < t < *t*_*r*_) where the internal pressure was linearly changed up to a certain value over the rise time *t*_*r*_ resulting in gradual displacements and change of height at the top surface *δ*(*t*). The difference between internal and external pressure induces pressure gradients across the cell boundaries and causes fluid to flow from outside to inside, resulting in bulk volumetric swelling/deswelling. For swelling *P*_*eff.*_ > 0 → *P*_out_ > *P*_in_ was considered, and for deswelling simulations *P*_*eff.*_ < 0 → *P*_out_ < *P*_in_ was considered. The poroelastic material domain was discretised using the quadratic quadrilateral CPE8P element and the sensitivity of the FE simulations to domain size and mesh element numbers were checked: a mesh convergence study was performed in the ramp phase by decreasing the mesh size until the estimated maximum displacement *δ*_*∞*_, at the top surface in the finer mesh, differed by less than 0.1% of the *δ*_*∞*_ in the coarser mesh. In the FE solver, a tolerance parameter for maximum *pore* pressure change (in our case pore pressure being equal to effective pressure as we do not explicitly model osmotic pressure) per increment was used. Because the pore pressure and fully relaxed maximum displacement *δ*_*∞*_ are the main effective output parameters of these simulations, the iteration convergence was achieved by decreasing the pore pressure rate change until the error in the maximum displacement *δ*_*∞*_ was minimized (Figure S2). For completeness, we also compared linear poroelasticity and neo-Hookean porohyperelasticity. As for the cell-related porohyperelastic parameters (*C*_10_, *D*_1_, *D*, *η*), they were taken from previous studies (Moeendarbary et al., 2013; Valero et al., 2016) and are summarized in Tables S1 and S2.

To computationally evaluate effects of cell volume changes on the poroelastic parameters (i.e. *E*, *D*), we conducted indentation simulations on central regions of a simple constrained geometry as shown in Figure 3. An infinitely rigid indenter of size *R* = 4.5μm was pressed into the simulated samples (control, swelled and shrunk cases) with a fast approach velocity. The force-indentation and force-relaxation curves were analyzed to extract the elastic modulus and poroelastic diffusion coefficient (see (Esteki et al., 2020) for detailed methodology and (Hu et al., 2010) for curve fitting procedures).

### Quantification and statistical analysis

Figures represent averaged or representative results of multiple independent experiments or simulations. The figure legends provide details concerning the number of independent experiments. Analyses were performed using data analysis toolbox in Microsoft Excel or Origin.

## Data Availability

Original/source data in this study is available upon request. All original code is available in this papers supplemental information. Any additional information required to reanalyze the data reported in this study is available from the lead contact upon request.
