# Supplementary material for: Poroelastic osmoregulation of living cell volume
Source: iScience. 2021 Nov 22;24(12):103482. doi: 10.1016/j.isci.2021.103482 (PMC8649806; doi:10.1016/j.isci.2021.103482)
Supplement: Document S1. Figures S1–S13 and Tables S1 and S2 [file mmc1.pdf]

iScience, Volume *24*

Supplemental information

Poroelastic osmoregulation of living cell volume

Mohammad Hadi Esteki, Andrea Malandrino, Ali Akbar Alemrajabi, Graham K. Sheridan, Guillaume Charras, and Emad Moeendarbary

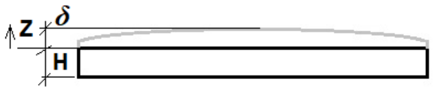

Fig. S1 Schematic illustration of a constrained thin hydrogel layer, related to STAR Methods. The initial state is assumed to be isotropically swollen from the dry state with the relevant geometrical parameters: displacement (δ), material thickness (H).

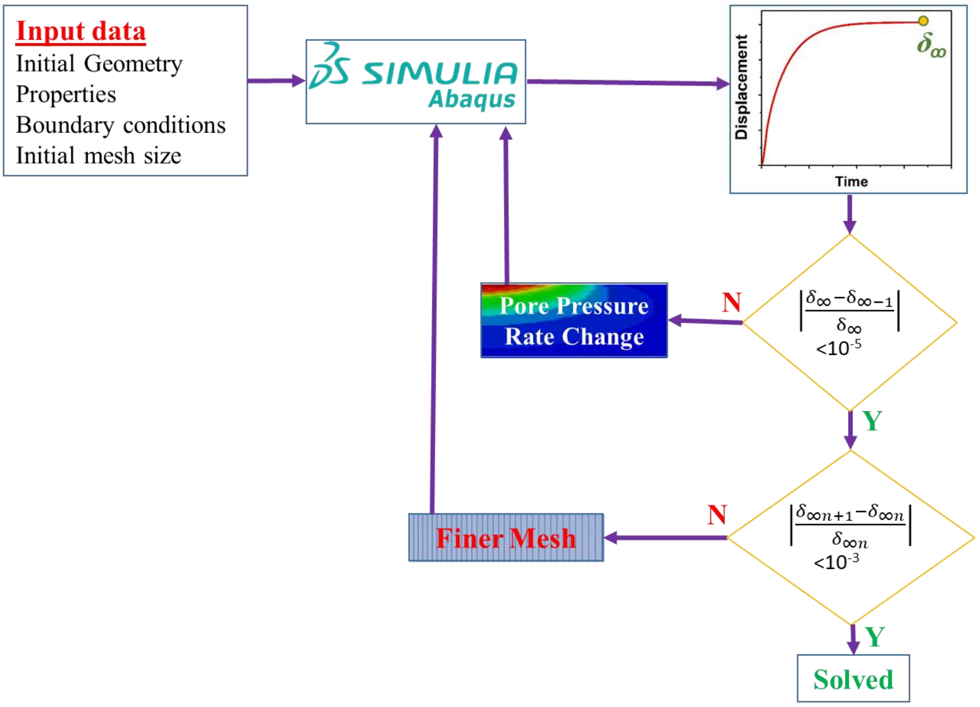

Fig. S2 FE methodology flowchart, related to STAR Methods. For every t, the maximum height displacement δ is calculated. Convergence of the finite element method program for each case is guaranteed by comparing the effective pressure rate and the maximum displacement (δ_∞) error in their independent loops.

Table S1. Poroelastic properties applied in FE model(Moeendarbary *et al.*, 2013), related to Fig. 3

Geometry	Shear modulus, G (kPa)	Diffusivity, D ($\mu m^2/s$)	Poisson's ratio, υ	Fluid viscosity, $\eta(N.s/m^2)$
Rectangular	0.19	30	0.3	1.0×10^{-3}

Table S2. Porohyperelastic properties of neo-Hookean model in FE model(Moeendarbary *et al.*, 2013; Valero *et al.*, 2016), related to Fig.4

Geometry	Material constant, C_{10} (kPa)	Material constant, D_1 $(kPa)^{-1}$	Diffusivity, D ($\mu m^2/s$)	Fluid viscosity, $\eta(N.s/m^2)$
Rectangular	0.096	4.8	30	1.0×10^{-3}
Cell	0.19	2.4	10	1.0×10^{-3}

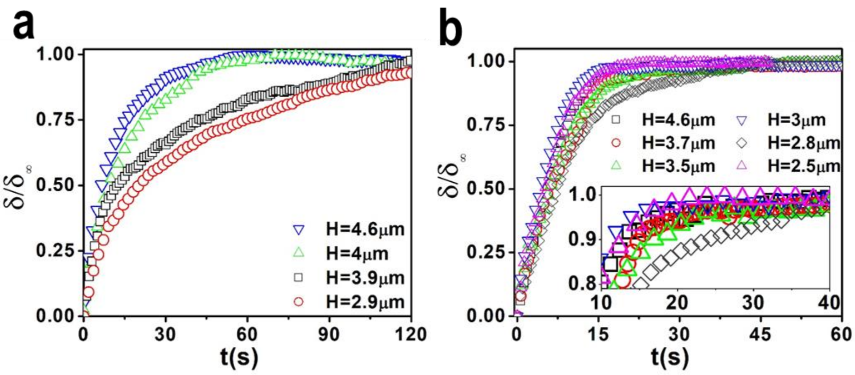

Fig. S3 FE Normalized displacement curves, related to Figs. 2b,c. (a,b) Normalization of swelling (Fig. 2b) and deswelling (Fig. 2c) curves considering $\frac{\delta}{\delta_\infty}$ for y-axis.

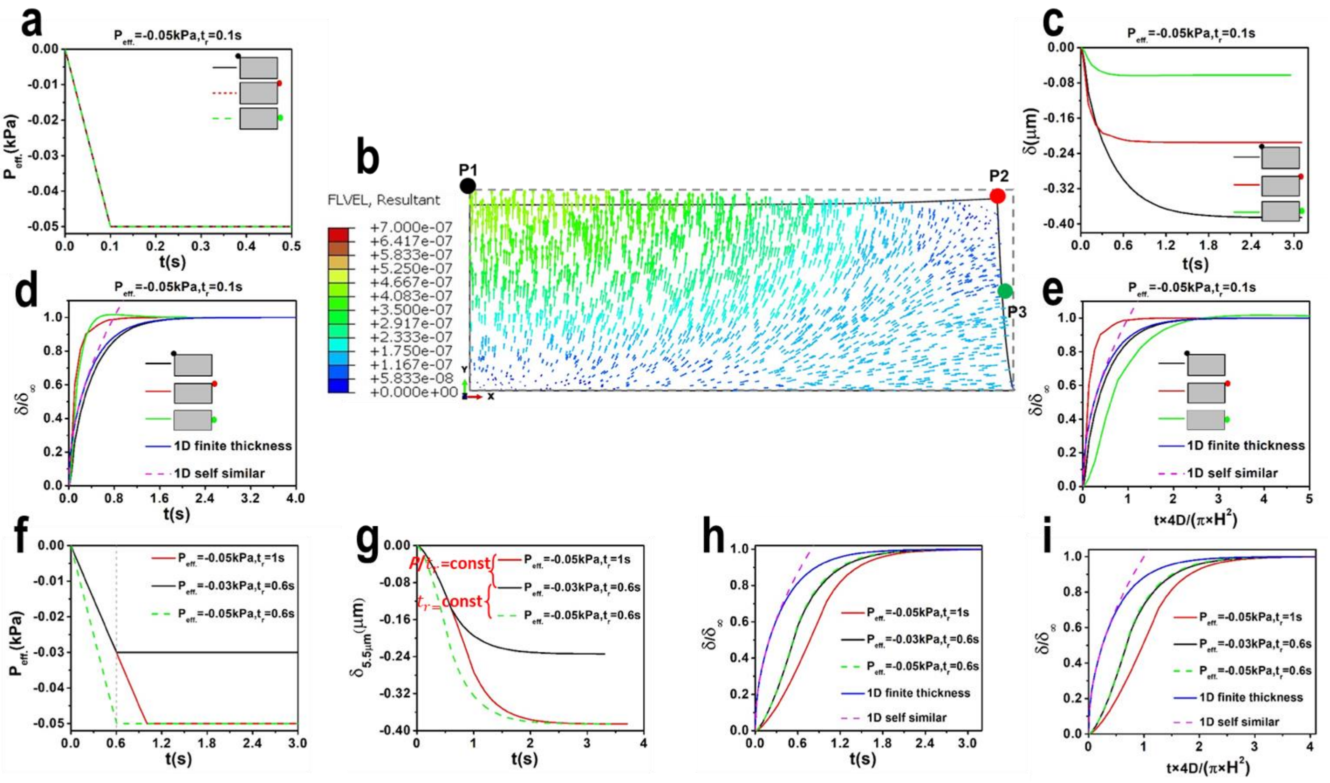

Fig. S4 Effects of spatial location, rise time, effective pressure for constrained deswelling of a simple geometry investigated by FE linear poroelastic simulations, related to Fig. 3. (a) Linear increase of effective pressure from 0 to $P_{eff.}$ =-0.05 kPa over rise time $t_r = 0.1$ s. **(b)** The deswelling type deformation of the hydrogel due to application of pressure difference and outflowing of water $P_{eff.} < 0 \rightarrow P_{in} > P_{out}$. Vertical displacements of specific nodes located at different positions P1, P2 and P3 were studied. Dash and solid lines indicate non-deformed and deformed shapes respectively. Fluid velocity field distributed non-uniformly at boundaries and within the domain induce a non-uniform deswelling behavior across the geometry. **(c)** Plot of vertical δ over time considering ramp in pressure indicated in (a). **(d)** Normalization of (c) considering $\frac{\delta}{\delta_\infty}$ for y-axis and plots of 1D finite thickness and self-similar solutions. **(e)** Normalization of (c) considering $\tau = \frac{4Dt}{\pi H^2}$ for x-axis and $\frac{\delta}{\delta_\infty}$ for y-axis. **(f)** Application of ramp with two different $P_{eff.}$ and two different t_r. **(g)** Considering conditions in (f), the plots of vertical displacement δ vs time were extracted for position P1. **(h)** Normalization of (g) considering $\frac{\delta}{\delta_\infty}$ for y-axis and plots of 1D finite thickness and self-similar solutions. **(i)** Normalization of both x and y axis led to the overlaying of the curves when $\left(\frac{P_{eff.}}{\delta_\infty}\right)_1 = \left(\frac{P_{eff.}}{\delta_\infty}\right)_2$. The analytical solutions cannot fit any of the normalized curves.

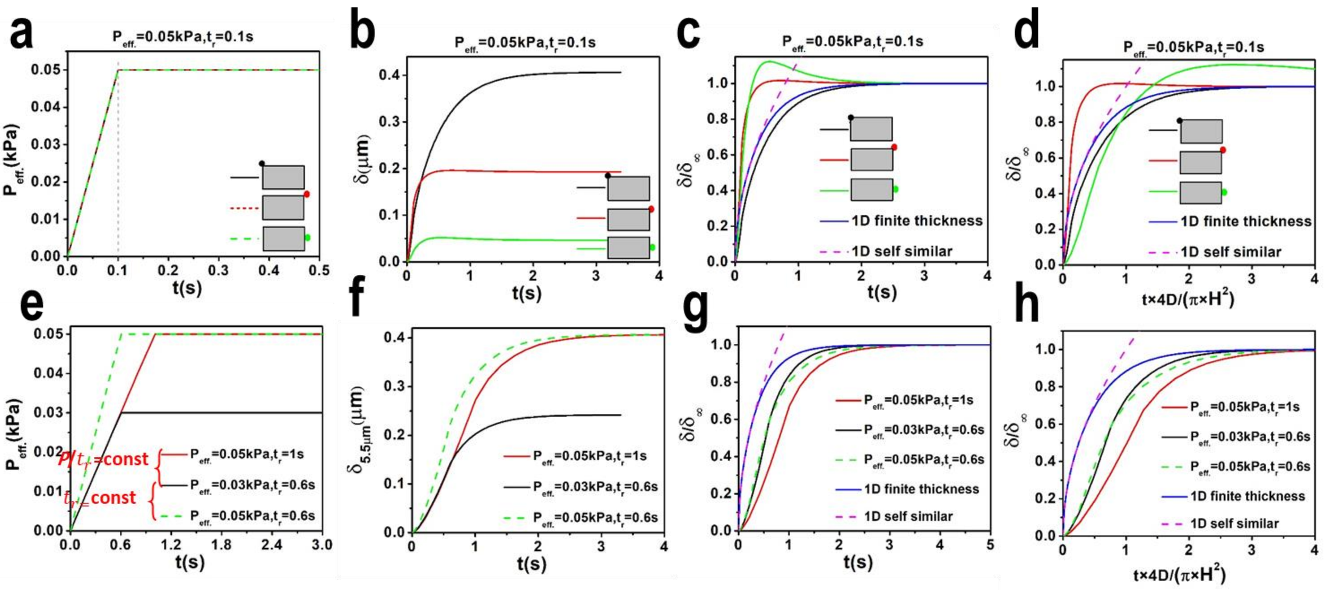

Fig. S5 Effects of spatial location, rise time, effective pressure for constrained swelling of a simple geometry investigated by FE porohyperelastic simulations, related to Fig. 3. (a) Linear increase of effective pressure from 0 to $P_{eff.}$ =0.05 kPa over rise time $t_r = 0.1$ s. **(b)** Plot of vertical δ over time considering ramp in pressure indicated in (a) at different positions P1, P2 and P3. **(c)** Normalization of (b) considering $\frac{\delta}{\delta_\infty}$ for y-axis and plots of 1D finite thickness and self-similar solutions. **(d)** Normalization of (c) considering $\tau = \frac{4Dt}{\pi H^2}$ for x-axis and $\frac{\delta}{\delta_\infty}$ for y-axis. **(e)** Application of ramp with two different $P_{eff.}$ and two different t_r. **(f)** Considering conditions in (e), the plots of vertical displacement δ vs time were extracted for position P1. **(g)** Normalization of (f) considering $\frac{\delta}{\delta_\infty}$ for y-axis and plots of 1D finite thickness and self-similar solutions. **(h)** Normalization of both x and y axis led to the overlaying of the curves when $\left(\frac{P_{eff.}}{\delta_\infty}\right)_1 = \left(\frac{P_{eff.}}{\delta_\infty}\right)_2$. The analytical solutions cannot fit any of the normalized curves.

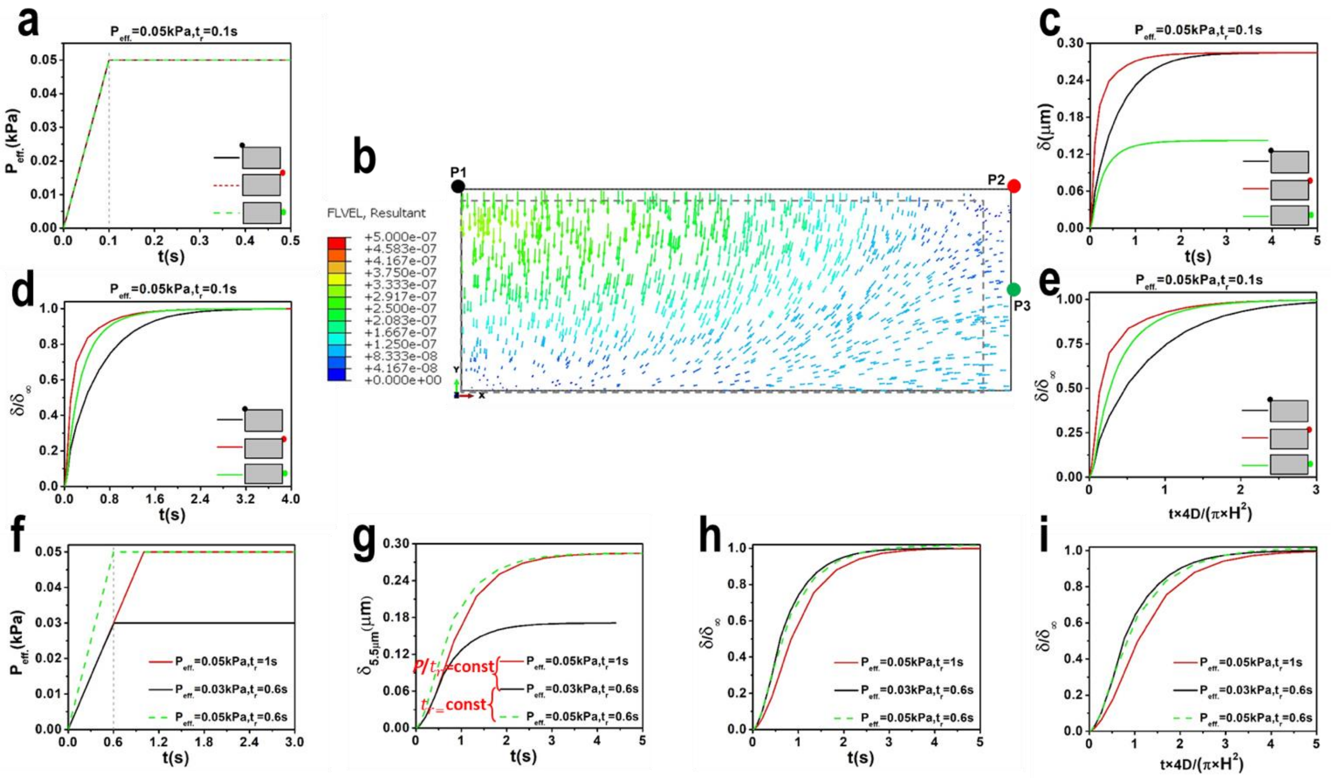

Fig. S6 Effects of spatial location, rise time, effective pressure for free swelling of a simple geometry investigated by FE linear poroelastic simulations, related to Fig. 3. (a) Linear increase of effective pressure from 0 to $P_{eff.}$ =0.05 kPa over rise time $t_r = 0.1$ s. **(b)** The swelling type deformation of the hydrogel due to application of pressure difference and inflowing of water $P_{eff.} > 0 \rightarrow P_{in} < P_{out}$. Vertical displacements of specific nodes located at different positions P1, P2 and P3 were studied. Dash and solid lines indicate non-deformed and deformed shapes respectively. Fluid velocity field distributed non-uniformly at boundaries and within the domain induce a non-uniform swelling behavior across the geometry. **(c)** Plot of vertical δ over time considering ramp in pressure indicated in (a). **(d)** Normalization of (c) considering $\frac{\delta}{\delta_\infty}$ for y-axis. **(e)** Normalization of (c) considering $\tau = \frac{4Dt}{\pi H^2}$ for x-axis and $\frac{\delta}{\delta_\infty}$ for y-axis. **(f)** Application of ramp with two different $P_{eff.}$ and two different t_r. **(g)** Considering conditions in (f), the plots of vertical displacement δ vs time were extracted for position P1. **(h)** Normalization of (g) considering $\frac{\delta}{\delta_\infty}$ for y-axis and plots. **(i)** Normalization of (g) considering $\tau = \frac{4Dt}{\pi H^2}$ for x-axis and $\frac{\delta}{\delta_\infty}$ for y-axis did not lead to the overlaying of the curves when $\left(\frac{P_{eff.}}{\delta_\infty}\right)_1 = \left(\frac{P_{eff.}}{\delta_\infty}\right)_2$.

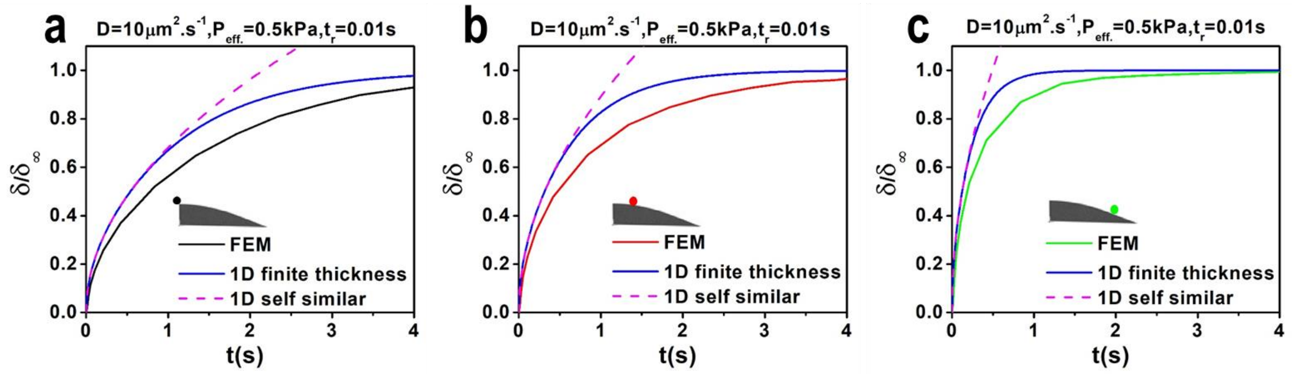

Fig. S7 Effects of spatial location for constrained swelling of a realistic cellular geometry investigated by FE porohyperelastic simulations, related to Fig. 4. 1D finite thickness and self-similar solutions comparing with normalized displacement curves of FEM (Fig. 4d) for **(a)** H=5 µm, **(b)** H=4 µm, **(c)** H=2.5 µm. different swelling behavior in different P1, P2 and P3 positions because of the cell geometry and boundary conditions.

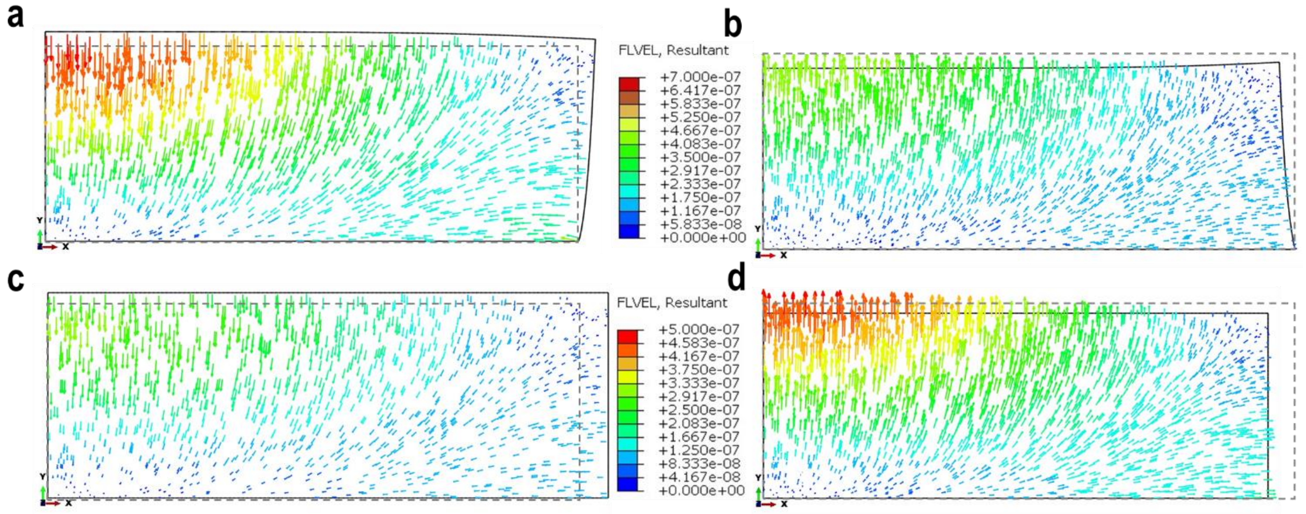

Fig S8. Simulated deformation and fluid velocity (mm.s^{-1}) field of the poroelastic hydrogel, related to Fig. 3. (a,b) Constrained swelling/deswelling and **(c,d)** free swelling/deswelling of the poroelastic FE model Under effective pressure of 0.05 kPa (swelling) and -0.05 kPa (deswelling) at t=0.1 s. The deformation of the hydrogel (solid line) due to positive effective pressure increases which cause to inflow and swelling (a,c) and the deformation of the hydrogel due to negative effective pressure decreases which cause to outflow of the fluid and deswelling (b,d). Dash lines indicate the non-deformaed hydrogel with thin disc geometry. Fluid velocity field distributed non-uniformly at boundaries and within the domain induce a non-uniform swelling/deswelling behavior across the geometry.

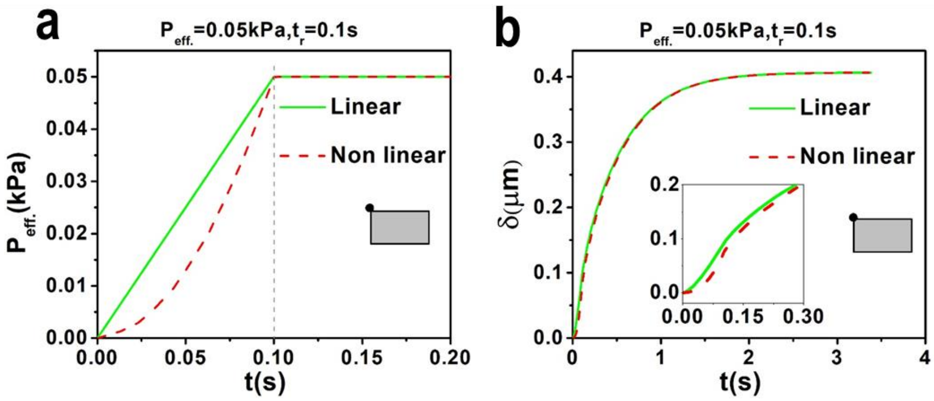

Fig S9. Effects of nonlinear pressure change for constrained swelling of a simple geometry investigated by FE linear poroelastic simulations, related to Fig. 3. (a) Linear and nonlinear increase of effective pressure from 0 to $P_{eff.}$ =0.05 kPa over a rise time t_r=0.1s. **(b)** Plot of vertical δ over time considering linear and nonlinear of the ramp in pressure indicated in (a).

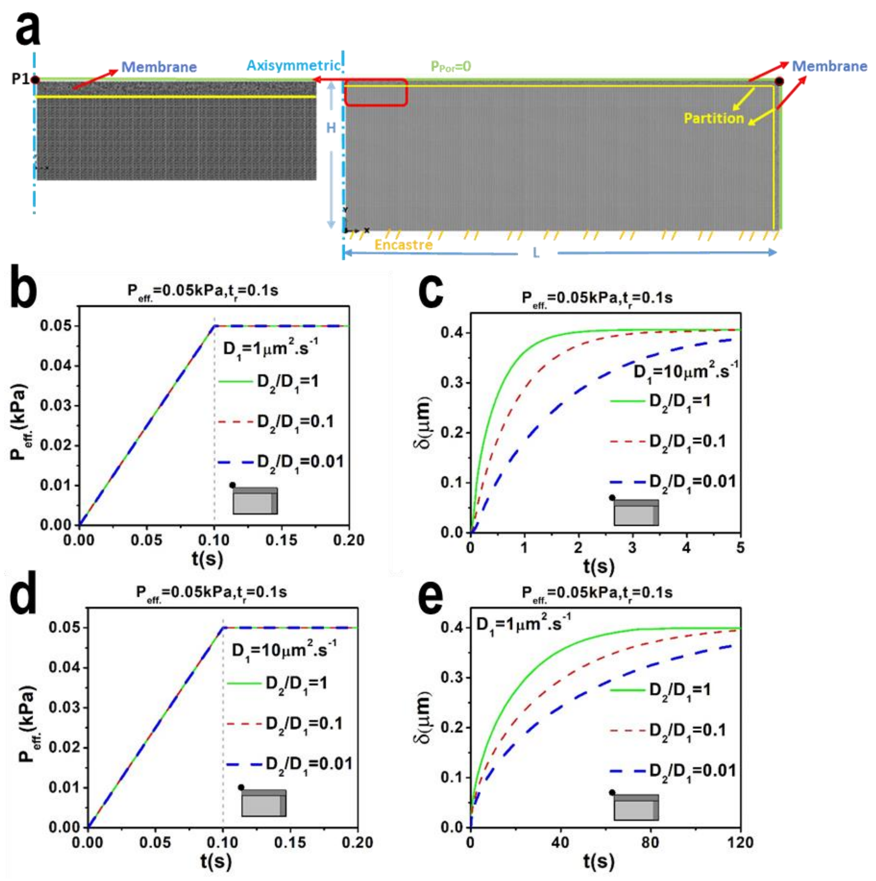

Fig S10. Effects of membrane consideration for constrained swelling of a simple geometry investigated by FE linear poroelastic simulations, related to Fig. 3. (a) Axisymmetric FE model of a double layer thin disk with thickness H and length L **(b,d)** Linear increase of effective pressure from 0 to $P_{eff.}$ =0.05 kPa over a rise time t_r=0.1s for different diffusion coefficients of base layer. **(c)** Plot of vertical δ over time considering the ramp in pressure indicated in (b) with D_1=10µm^2.s^{-1} for base layer and different diffusion coefficients of the membrane. **(e)** Plot of vertical δ over time considering the ramp in pressure indicated in (d) with D_1=1µm^2.s^{-1} for base layer and different diffusion coefficients of the membrane.

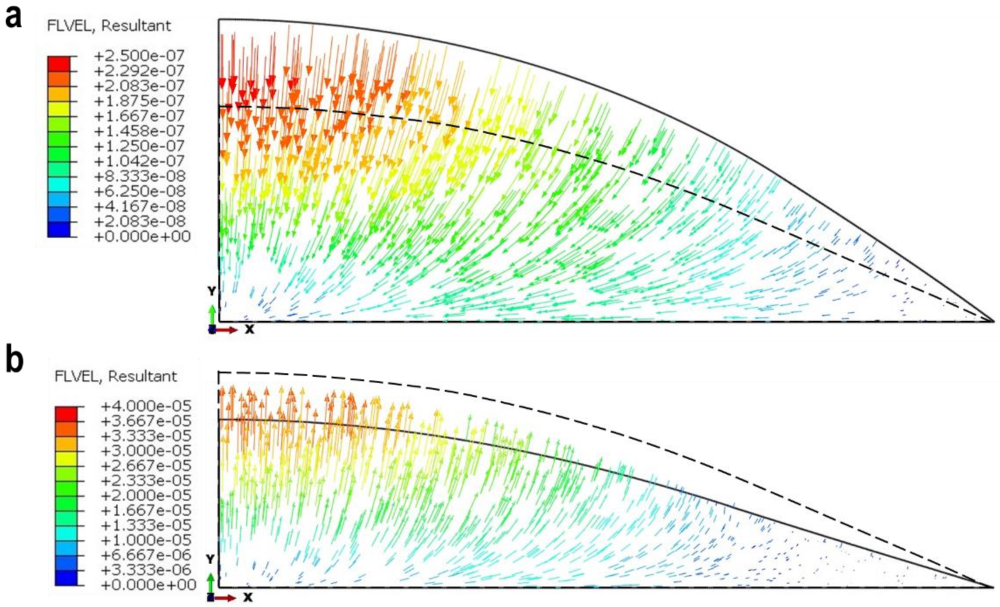

Fig S11. Simulated deformation and fluid velocity (mm.s-1) field of the porohyperelastic cell, related to Fig. 4. (a) Constrained cell swelling and **(b)** deswelling of the porohyperelastic FE model. Under effective pressure of 0.5 kPa (swelling) and -0.5 kPa (deswelling) at t=0.1 s. The cell swells due to application of pressure changes across cell membrane and entering of water into cell interior. Dash and solid lines indicate non-deformed and deformed shapes respectively. Fluid velocity field is also show with color arrows.

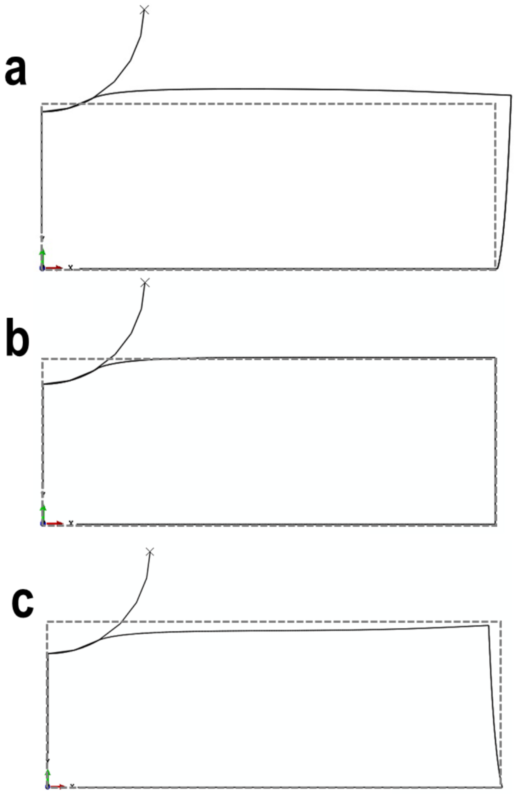

Fig S12. Simulated deformation of the porohyperelastic hydrogel within indentation test, related to STAR Methods. Porohyperelastic indentation tests were simulated on **(a)** swelled, **(b)** control and **(c)** shrunk cases. (a,c) Constrained swelling/deswelling under effective pressure of 0.05 kPa (swelling) and -0.05 kPa (deswelling) and (b) the porohyperelastic FE model of indentation on the non-swelling/deswelling surface by methodology has been detailed in (Esteki *et al.*, 2020). The deformation of the hydrogel (solid line) due to (a) positive effective pressure increases which cause to inflow and swelling and the deformation of the hydrogel due to (c) negative effective pressure decreases which cause to outflow of the fluid and deswelling. Dash lines indicate the non-deformed state.

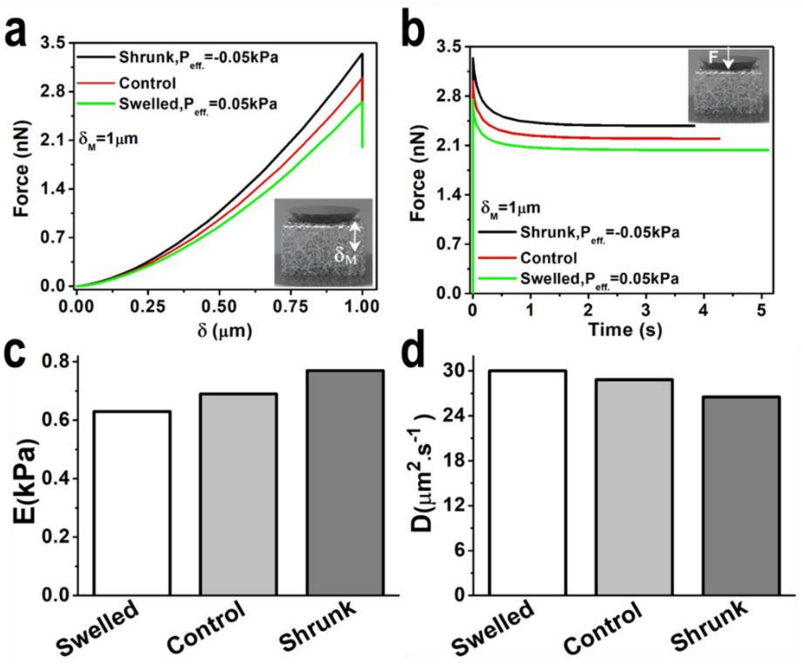

Fig S13. Effect of swelling and deswelling on the poroelastic parameters, related to STAR Methods. Porohyperelastic indentation tests were simulated on controlled, swelled and deswelled cases. Force-indentation **(a)** and force-relaxation **(b)** curves generated from FEM considering fast indentation velocity and a maximum indentation depth of δ_M=1μm. The maximum displacement δ_M achieved at infinitely fast indentation velocity (V~∞ or t_r~0) (Esteki *et al.*, 2020) **(c)** The diffusion coefficient and **(d)** elastic modulus obtained from indentation simulations on swelled, control and shrank cell states as in Fig.S12 and using the methodology as described in (Hu *et al.*, 2010).
